# Aromatic l-amino acid decarboxylase deficiency: a patient-derived neuronal model for precision therapies

**DOI:** 10.1093/brain/awab123

**Published:** 2021-03-18

**Authors:** Giada Rossignoli, Karolin Krämer, Eleonora Lugarà, Haya Alrashidi, Simon Pope, Carmen De La Fuente Barrigon, Katy Barwick, Giovanni Bisello, Joanne Ng, John Counsell, Gabriele Lignani, Simon J R Heales, Mariarita Bertoldi, Serena Barral, Manju A Kurian

**Affiliations:** 1 Developmental Neurosciences, GOS Institute of Child Health, University College London, London WC1N 1EH, UK; 2 Biological Chemistry, NBM Department, University of Verona, 37134 Verona, Italy; 3 Clinical and Experimental Epilepsy, Queen Square Institute of Neurology, University College London, London WC1N 3BG, UK; 4 Genetics and Genomic Medicine, GOS Institute of Child Health, University College London, London WC1N 1EH, UK; 5 Neurometabolic Unit, National Hospital for Neurology and Neurosurgery, Queen Square, London WC1N 3BG, UK; 6 Gene Transfer Technology Group, EGA-Institute for Women's Health, University College London, London WC1E 6HU, UK; 7 Centre for Inborn Errors of Metabolism, GOS Institute of Child Health, UniversCity College London, London WC1N 1EH, UK; 8 Department of Neurology, Great Ormond Street Hospital, London WC1N 3JH, UK

**Keywords:** induced pluripotent stem cells, dopaminergic neurons, aromatic l-amino acid decarboxylase deficiency, neurodevelopment, personalized medicine

## Abstract

Aromatic l-amino acid decarboxylase (AADC) deficiency is a complex inherited neurological disorder of monoamine synthesis which results in dopamine and serotonin deficiency. The majority of affected individuals have variable, though often severe cognitive and motor delay, with a complex movement disorder and high risk of premature mortality. For most, standard pharmacological treatment provides only limited clinical benefit. Promising gene therapy approaches are emerging, though may not be either suitable or easily accessible for all patients. To characterize the underlying disease pathophysiology and guide precision therapies, we generated a patient-derived midbrain dopaminergic neuronal model of AADC deficiency from induced pluripotent stem cells. The neuronal model recapitulates key disease features, including absent AADC enzyme activity and dysregulated dopamine metabolism. We observed developmental defects affecting synaptic maturation and neuronal electrical properties, which were improved by lentiviral gene therapy. Bioinformatic and biochemical analyses on recombinant AADC predicted that the activity of one variant could be improved by l-3,4-dihydroxyphenylalanine (l-DOPA) administration; this hypothesis was corroborated in the patient-derived neuronal model, where l-DOPA treatment leads to amelioration of dopamine metabolites. Our study has shown that patient-derived disease modelling provides further insight into the neurodevelopmental sequelae of AADC deficiency, as well as a robust platform to investigate and develop personalized therapeutic approaches.

## Teaser

Rossignoli *et al*. develop the first humanized neuronal model of AADC deficiency. They use this patient-derived neuronal system to elucidate disease mechanisms, and in particular to better define neurodevelopmental features, as well as to test precision therapy approaches.

## Introduction

Neurodevelopmental processes are commonly disrupted in the vast majority of inborn errors of metabolism, resulting in a wide repertoire of clinical manifestations from severe cognitive, neuropsychiatric, and motor problems to more subtle learning difficulties.^1^ Aromatic l-amino acid decarboxylase (AADC) deficiency is a rare inborn error of neurotransmitter metabolism due to bi-allelic mutations in *DDC*, which encodes the enzyme that catalyses the final step of serotonin and dopamine synthesis.^2^ The resultant enzyme deficiency leads to combined serotonin and catecholamine (dopamine, norepinephrine, epinephrine) deficiency.^3^ Although there is a wide phenotypic spectrum,^4^^,^^5^ the majority of affected patients show many of the typical features seen in recessively inherited, early-onset neurotransmitter disorders,^6^ including severe global neurodevelopmental delay, oculogyric crises, a complex movement disorder (characterized by central and peripheral hypotonia, commonly with features of dystonia/chorea) and symptoms of dysautonomia, as well as secondary gastrointestinal, respiratory and orthopaedic complications.^7^^,^^8^ As a result, most patients have significant disability and high risk of premature mortality. AADC deficiency is associated with a characteristic CSF monoamine profile, with reduced 5-hydroxyindoleacetic acid (5-HIAA), homovanillic acid (HVA), and 3,4-dihydroxyphenylacetic acid (DOPAC), and a concomitant increase in 5-hydroxytryptophan (5-HTP), l-3,4-dihydroxyphenylalanine (l-DOPA), and 3-*O*-methyldopa (3-OMD). Definitive diagnosis is ideally achieved by confirming a decrease or absence of plasma AADC enzymatic activity, and *DDC* gene sequencing. To date, there are no clear correlations between patient genotype, CSF monoamine profile, AADC enzyme activity and phenotype.

A recently published consensus guideline outlines recommendations for the diagnosis and management of AADC deficiency.^8^ Pharmacological therapy provides some, though often limited, clinical benefit and patients often show variable drug response. It has been postulated that the variability in disease severity and medication response may be partly attributed to genotype^9^^,^^10^ and as a result, a number of studies have focused on characterizing the underlying molecular defects caused by different pathogenic variants.^11-15^ More recently, promising gene therapy approaches are emerging for AADC deficiency, with a number of clinical trials evaluating the safety and efficacy of targeted intraparenchymal delivery of AAV2-based vectors.^16–18^ It is hoped that with time, these studies may clarify the effect of patient genotype, age at surgery, pretreatment motor function and target delivery site on overall therapeutic efficacy. Although early clinical studies on AADC gene therapy are encouraging, it is likely that this therapeutic strategy may not be either viable, suitable or easily accessible for a proportion of patients. Moreover, with advances in diagnostic testing, the global incidence and prevalence of AADC deficiency continues to increase,^19^ and the need for alternative precision therapies is increasingly apparent.

Recently, patient-derived cellular models of neurodevelopmental disorders have proven to be a valuable experimental system to unravel disease mechanisms and test novel therapeutic strategies with translational potential.^20^ As such, we have developed a humanized neuronal model of AADC deficiency, by reprogramming patient fibroblasts into induced pluripotent stem cells (iPSCs) for differentiation into midbrain dopaminergic (mDA) neurons. This model system has allowed us to gain further insight into the neurodevelopmental consequences of AADC deficiency, with effects on synaptic maturation and neuronal function. Moreover, it has also provided a suitable platform to evaluate the effects of precision medicine approaches at a cellular level, demonstrating the potential for rational development of patient-specific strategies in such rare monogenic disorders.

## Materials and methods

### Induced pluripotent stem cell generation and maintenance

Generation of iPSCs from patient dermal fibroblasts was approved by the Local Research Ethics Committee (Reference 13/LO/0171). Written informed consent was obtained from all patients. Age-matched healthy control fibroblasts were collected from the MRC Centre for Neuromuscular Disorders Biobank. Patient fibroblasts were isolated from skin biopsies and maintained in DMEM (Gibco), 10% foetal bovine serum (Gibco), 2 mM l-glutamine (Gibco), 1% MEM non-essential amino acids (Gibco), and 1% penicillin/streptomycin (P/S, Gibco), and tested for mycoplasma contamination. Reprogramming was performed using the commercially available CytoTune^®^-iPS 2.0 Sendai Reprogramming kit (Invitrogen), following manufacturer's instructions. Fibroblast were transduced at 80% confluence (1–1.5 × 10^5^ cells/well). After 6 days, infected cells were harvested with TrypLE^TM^ (Invitrogen) and 8000 cells/well were seeded onto gamma-irradiated mouse embryonic fibroblasts. After 24 h, cells were cultured into KO-DMEM (Gibco), 20% serum replacement (Gibco), 2 mM l-glutamine, 50 µM 2-mercaptoethanol, 1% MEM non-essential amino acids, 1% P/S, and 10 ng/ml basic fibroblast growth factor (Gibco). 13 days post-transfection, cells were cultured in gamma-irradiated mouse embryonic fibroblasts-conditioned medium. Around Day 30 post-transduction, 8–10 independent colonies with iPSCs-like morphology were collected and expanded using ReLeSR^TM^ (Stemcell Technologies). Between passage 15 and 20, three colonies were converted to mTeSR1 medium (Stemcell Technologies) on Matrigel^®^ (Corning^®^) coated plates. Derived iPSC lines were maintained in the mTeSR1/Matrigel system, regularly passaged with EDTA, 0.02% solution (Sigma-Aldrich) and again tested for mycoplasma infection, as previously. Two iPSC lines for each patient (Patients 1-04, 1-10, 2-01 and 2-06) and the age-matched healthy control (Control-05 and Control-03) were characterized at the iPSCs stage and further differentiated into mDA neurons to exclude clonal variability. Given the relative homogeneity reported in clonal lines with respect to transcriptome, growth, and capability of germ layer formation,^21^^,^^22^ one clone per patient (Patient 1-04; Patient 2-01) and age-matched healthy control (Control-05) were then used for downstream experiments.

### Differentiation of induced pluripotent stem cells into midbrain dopaminergic neurons

IPSCs were differentiated into mDA neurons as previously described.^23^ Briefly, iPSCs were harvested using TrypLE^TM^ (Invitrogen), and plated onto non-adherent bacterial dishes at a concentration of 1.5 × 10^5^ cells/cm^2^ in DMEM/F12:Neurobasal^TM^ (1:1), N2 (1:100) and B27 minus vitamin A (1:50) supplements (Invitrogen), 2 mM l-glutamine and ROCK-inhibitor for the first 2 days. Embryoid bodies were plated at Day 4 onto polyornithine (PO; 15 μg/ml; Sigma), fibronectin (FN; 5 μg/ml Gibco) and laminin (LN; 5 μg/ml; Sigma) coated dishes in DMEM/F12:Neurobasal^TM^ (1:1), N2 (1:200), B27 minus vitamin A (1:100), 2 mM l-glutamine. From Day 0 to Day 9, medium was supplemented with: 10 μM SB431542 (Tocris Bioscience), 100 nM LDN193189 (Stemgent Inc), 0.8 µM CHIR99021 (Tocris Biosceince) and 100 ng/ml hSHH-C24-II (R&D Systems). On Day 2, 0.5 μM purmorphamine (Cambridge Bioscience) was added. SB431542 was withdrawn on Day 6. On Day 11, cells were either processed for mDA precursors analysis or harvested with Accumax and replated on PO/FN/LN coated dishes in droplets of 1–1.5 × 10^4^ cells/µl in Neurobasal^TM^/B27 minus vitamin A (1:50), 2 mM l-glutamine, 0.2 mM ascorbic acid and 20 ng/ml BDNF (Miltenyi Biotech). On Day 14 of differentiation, 0.5 mM dibutyryl cAMP (Sigma-Aldrich) and 20 ng/ml GDNF (Miltenyi Biotech) were added. On Day 30 of differentiation, cells were replated as describe above onto PO/FN/LN coated dishes or Lab-Tek^TM^ slides (Nunc^TM^), and γ-secretase inhibitor DAPT (10 μM, Tocris) was added until final differentiation at Day 65. Cells were then harvested or processed for further analysis.

### AADC activity assay

AADC enzyme assay was performed using the refined method developed in Allen^24^ from Hyland and Clayton.^25^ Neuronal cultures at Day 65 in phenol red-free medium were harvested and lysed by snap freezing twice in liquid nitrogen in 100 µl of 10 mM Tris pH 7.4 (Sigma-Aldrich), 1 mM EDTA, 320 mM sucrose (Sigma-Aldrich) and protease inhibitor cocktail (Roche). Cell lysate (50 μl) was incubated with 70 μM pyridoxal 5′-phosphate (PLP, Sigma-Aldrich) in assay buffer composed by 500 mM sodium phosphate pH 7.0, 0.167 mM EDTA, and 39 mM dithiothreitol (Sigma-Aldrich) for 120 min at 37°C, and subsequently 2 mM final concentration of l-DOPA (Sigma-Aldrich) was added and incubated for 20 min at 37°C. The reaction was stopped with 250 μl of 0.8 M perchloric acid (final concentration 0.4 M) for 10 min at room temperature and centrifuged at 12 000*g* for 5 min at 4°C. A substrate blank with no l-DOPA and a sample blank without cell lysate were performed for each sample. Dopamine in the supernatant was then quantified by high performance liquid chromatography (HPLC).

### High performance liquid chromatography for quantification of activity assay and metabolic profile

Dopamine produced in the activity assay was separated by reverse-phase HPLC using a HiQSil C18 column 250 × 4.6 mm (Kya Technologies) and detected by coulometric electrochemical detection using a Coulochem^®^ III detector (ESA) with 5010 analytical cell (ESA) setting the detector electrode at 350 mV and the screening electrode at 20 mV. The mobile phase consisted of 50 mM sodium phosphate pH 3.6, 5 mM octaensulfonic acid, 67 μM EDTA, 43 mM orthophosphoric acid and 230 ml/l methanol diluted in 18.2 Ω HPLC grade water, at a flow rate of 1.2 ml/min at 25°C. Dopamine was quantified with Azur software package using a 1000 nM external standard and enzymatic activity was expressed as pmol/min/mg protein.

HPLC analysis of metabolic profile in derived mature cultures was performed on the phenol red-free medium incubated for 48 h on Day 65 mDA neurons. 1:1 medium was mixed with perchloric acid to a final concentration of 0.4 M, incubated 10 min at 4°C in the dark, centrifuged at 12 000*g* for 5 min at 4°C, and supernatant was collected for analysis by HPLC.^26^ Metabolites were separated by reverse-phase HPLC using a C18HS column 250 mm × 4.5 mm (Kromatek) and detected by coulometric electrochemical detection using a Coulochem^®^ III detector (ESA) with 5010A analytical cell (Thermo Fisher Scientific) setting the detector electrode at 450 mV and the screening electrode at 20 mV. Mobile phase consisted of 20 mM sodium acetate trihydrate pH 3.45, 12.5 mM citric acid monohydrate, 100 μM EDTA, 3.35 mM octaensulfonic acid and 16% methanol diluted in 18.2 Ω HPLC grade water, at a flow rate of 1.5 ml/min at 27°C. Metabolites were quantified with EZChrom Elite^TM^ chromatography software (JASCO) using a 500 nM external standard mixture, and expressed as pmol/mg protein.

### Bulk RNA sequencing analysis

Total RNA was isolated using the RNeasy^®^ mini kit (Qiagen) following manufacturer’s instructions. RNA libraries were prepared from 100 ng of total RNA using KAPA mRNA HyperPrep kit (Roche) according to the manufacturer’s protocol and sequenced with Illumina NextSeq 500 Mid Output 75 bp paired-end (∼22 M reads/sample). FASTQ obtained files were uploaded to Galaxy web platform, and the public server at usegalaxy.org was used for downstream analyses.^27^ FASTQ files were filtered with Trimmomatic (v.0.38), with SLIDINGWINDOW trimming and low quality (phread score <20) reads filter.^28^ Obtained reads were mapped to human reference genome (GRCh38) with HISAT2 (v.2.1.0).^29^ Fragments counts for genes were extracted with featureCounts (v.1.6.4) excluding duplicates, multimapping reads and chimeric fragments.^30^ Differential gene expression was analysed using edgeR (v.3.24.1), filtering low counts at 0.35 minimum counts per million, in at least three samples,^31^ and comparing disease status (patients versus control) and disease-specific genotype (Patient 2 versus Patient 1). Differentially expressed genes (DEGs) with a *P*-value < 0.05 and absolute fold change >2 were considered as statistically significant. Heat maps were generated from the row-scaled *z*-score of DEGs normalized counts obtained by EdgeR with complete-linkage Euclidean hierarchical clustering. Gene ontology (GO) enrichment analyses were performed using ShinyGO v0.61^32^ for biological process, and ClueGO v.2.5.7^33^ for cellular component and molecular function enrichments and groupings, with Benjamini-Hochberg *P*-value correction of false discovery rate (FDR) < 0.05 for statistical significance. Results from the expression analysis along with the raw sequence data were deposited in GEO (Gene Expression Omnibus), under accession GSE153990.

### Electrophysiology

Current-clamp recordings were performed on neurons at Day 65 of differentiation. The internal solution contained 135 mM K-gluconate, 4 mM KCl, 10 mM HEPES, 4 mM Mg-ATP, 0.3 mM Na-GTP, and 10 mM of Na_2_-phosphocreatine, at pH 7.3 and mOsm 291–295. The recording extracellular solution contained 125 mM NaCl, 2.5 mM KCl, 2 mM MgCl_2_, 1.25 mM KH_2_PO_4_, 2 mM CaCl_2_, 30 mM glucose, and 25 mM of HEPES at pH 7.4. Experiments were performed at room temperature (22–24°C). Neurons with unstable resting potential (or >−50 mV), bridge-balance >20 MΩ and/or holding current >200 pA were discarded. Bridge balance compensation was applied in current clamp and the resting membrane potential was held at −70 mV. A current steps protocol was used to evoke action potentials injecting 250 ms long depolarizing current steps of increasing amplitude (Δ10 pA). Action potentials were triggered holding the neurons around −60 mV/−55 mV. Neurons with repetitive spontaneous action potentials and repetitive evoked action potentials were considered to be functional mature mDA neurons. Recordings were acquired using a Multiclamp 700A amplifier (Axon Instruments, Molecular Devices) at 10 kHz and filtered at 2 kHz (Bessel) using WinEDR (John Dempster, University of Strathclyde). Recordings were not corrected for liquid junction potentials. The approximate cell capacitance was computed as: *capacitance* *=* *tau/R_i_*, whereby the time constant tau was found by fitting a single exponential function to the time points where the membrane voltage was between 10% and 95% of the initial charging decay slope of a negative hyperpolarizing current step. Input resistance was calculated fitting ΔV/ΔI at two hyperpolarizing steps (−20 and −10 pA) and a positive one (+10 pA). Action potentials were identified when the voltage signal crossed 0 V. Spontaneous excitatory postsynaptic currents were recorded in a voltage clamp and automatically detected with a template-based algorithm using Clampfit (Molecular Devices).

### Treatment with l-DOPA and cytotoxicity assay

Neuronal cultures at Day 65 of differentiation were treated with 80 µM l-DOPA in phenol red-free medium for 24 h. The medium was subsequently removed and analysed by HPLC, as described above. Dead-cell protease release measurements were performed using CytoTox-Glo™ Cytotoxicity Assay (Promega) according to the manufacturer’s instructions.

### Statistical analysis

Two-tailed Student’s *t*-test for single comparisons and statistical one-way ANOVA followed by Tukey’s multiple comparisons test were performed using GraphPad Prism. Results are reported as mean ± standard error of the mean (SEM) from at least three independent biological replicates, the exact number of which is stated for each experiment in the figure legend. Significance levels were determined by *P*-value, and shown on graphs with asterisks. *P*-values are presented as *P = 0.05–0.01, **P = 0.01–0.001, and ***P < 0.001.

### Data availability

Data supporting the findings of this study are available from the corresponding authors, upon reasonable request.

## Results

### Patient-derived midbrain dopaminergic neurons show loss of AADC activity and dysregulated dopamine synthesis

Dermal fibroblasts were obtained from two patients with AADC deficiency (Table 1). Patient 1 (homozygous missense variant c.1039C>G, p.R347G) presented with classical infantile onset disease, with early hypotonia, oculogyric crises and neurodevelopmental delay.^15^ He is currently 6.5 years of age, and although he continues to make neurodevelopmental progress, remains non-ambulant and non-verbal. Patient 2 (compound heterozygous variants c.19C>T, p.Arg7*; c.299G>C, p.C100S) had a classical infantile-onset presentation of disease with severe global developmental delay, oculogyric crises and hypoglycaemia, but over time showed a positive response to therapy and had an overall milder disease course. Once AADC deficiency was diagnosed at 3.5 years of age, the instigation of dopaminergic medication and other specific AADC deficiency treatments were associated with neurodevelopmental progress; independent ambulation was achieved by 4.5 years and spoken language by 5.5 years. From 10 to 18 years, adjunct therapies were needed to combat side effects from long-term use of the original treatments to maintain basic motor and verbal function. Now aged 22 years, he has ongoing learning difficulties, mild motor impairments, behavioural issues, autistic traits and neuropsychiatric symptoms of anxiety and intermittent low mood. IPSC lines were generated from dermal fibroblasts of both patients and from an age-matched healthy individual (control). Sequencing of genomic DNA confirmed that patient iPSC lines retained their specific *DDC* mutation (Supplementary Fig. 1A). All iPSCs lines showed clearance of viral transgenes, genomic integrity (Supplementary Fig. 1B and C), and true pluripotency (Supplementary Fig. 2A–D). IPSCs were then differentiated into mDA neurons, and both patient and control iPSC lines showed similar differentiation efficiency. After 11 days of differentiation, all lines showed high levels of mDA progenitors and typical midbrain precursor gene expression profiles (Supplementary Fig. 3A–C). By 65 days of differentiation, both control and patient lines comparably matured into neurons, in particular with dopaminergic identity (Supplementary Fig. 4A and B). Both control and patient-derived neuronal cultures showed upregulation of midbrain-related genes (Supplementary Fig. 4C). Whole-cell patch clamp electrophysiology confirmed that iPSC-derived mDA neurons were functional and exhibited continuous and rhythmic pacemaker-like activity (Supplementary Fig. 4D). Derived neuronal cultures were almost devoid of serotonergic neurons, restricting all further analyses specifically to the mDA neuronal subtype (Supplementary Fig. 5A).

**Table 1:** Phenotype and genotype features of AADC deficiency patients

Patient number	Patient line	Clinical phenotype	Zygosity	Location of mutation	Type of mutation	Amino acid change
1	Patient 1-04 Patient 1-10	Oculogyric crises (frequent) Hypotonia Movement disorder Non-ambulant Autonomic features Neurodevelopmental delay Non-verbal	Homozygous	Exon 11	Missense	Arg347Gly
2	Patient 2-01 Patient 2-06	Oculogyric crises (infrequent) Mild motor disorder but achieved independent ambulation Neurodevelopmental delay Behavioural issues Autistic traits Psychiatric symptoms	Heterozygous	Exon 2	Nonsense	Arg7*
				Exon 3	Missense	Cys100Ser

**Figure 1:**
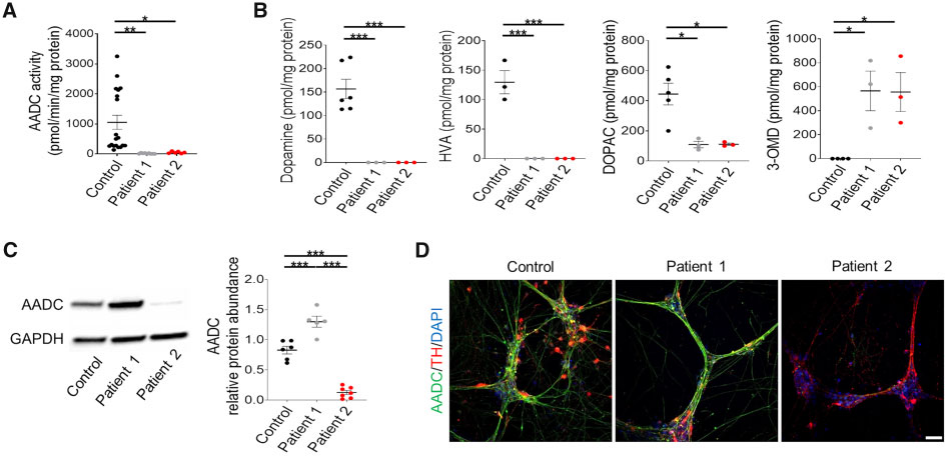
**Patient-derived neurons show loss of AADC enzymatic activity and dysregulated dopamine synthesis.** (**A**) AADC activity assay relative to total protein (*n* = 19, 9, and 6 for the control subject, Patient 1 and Patient 2, respectively). (**B**) HPLC detection of extracellular dopamine, HVA, DOPAC and 3-OMD in control, Patient 1 and Patient 2-derived neuronal cultures. Values are relative to total protein (*n* = 6, 3, 3; *n* = 3, 3, 3; *n* = 5, 3, 3; *n* = 4, 3, 3, respectively). (**C**) Immunoblot analysis for AADC protein in control, Patient 1 and Patient 2-derived neurons at Day 65 of differentiation. Quantification relative to loading control (GAPDH) (*n* = 6, 5, 7, respectively). (**D**) Representative images for AADC and TH immunofluorescence in derived neurons. Scale bar = 100 µm. Data are presented as mean ± SEM. *P < 0.05; **P < 0.01; ***P < 0.001, one-way ANOVA followed by Tukey’s multiple comparisons test.

We first investigated the effect of patient mutations on AADC enzyme activity and protein expression. Measurement of AADC activity showed significantly lower enzymatic function in patients when compared to control-derived neurons (Fig. 1A). HPLC analysis of extracellular metabolites showed a disease-specific absence of dopamine and HVA with significantly reduced levels of DOPAC. In contrast, 3-OMD, a downstream metabolite of the AADC substrate l-DOPA, was significantly increased in patient-derived neurons (Fig. 1B). Analysis of AADC protein levels showed an increase in Patient 1 neuronal cultures when compared to the control subject; in contrast, a significant reduction of AADC protein was detected in Patient 2 neuronal cultures (Fig. 1C, D and Supplementary Fig. 5B), in line with the second heterozygous early stop codon variant predicted to result in nonsense-mediated mRNA decay.

We then explored whether the aberrant AADC protein levels in patients could be linked to a difference in intrinsic protein stability. Recombinant AADC proteins were produced for the homozygous R347G variant (Patient 1) and C100S variant (Patient 2). Circular dichroism and dynamic light scattering analyses showed comparable values for both mutant and wild-type AADC protein, inferring similar intrinsic protein stability (Supplementary Table 1).

To investigate whether aberrant AADC protein levels related to *DDC* gene expression, quantitative RT-PCR studies were undertaken. In line with protein expression data, we observed a statistically significant increase in *DDC* expression in Patient 1 when compared to the control (Supplementary Fig. 5C). For Patient 2, we observed comparable levels of *DDC* expression to the control (Supplementary Fig. 5C), despite the predicted nonsense-mediated decay of a proportion of transcripts. We also observed an increase in tyrosine hydroxylase (TH) protein and gene expression in both patient lines when compared to the control (Supplementary Fig. 5D and E).

### AADC deficiency has mutation-specific effects on neuronal synaptic maturation and connectivity

**Figure 2:**
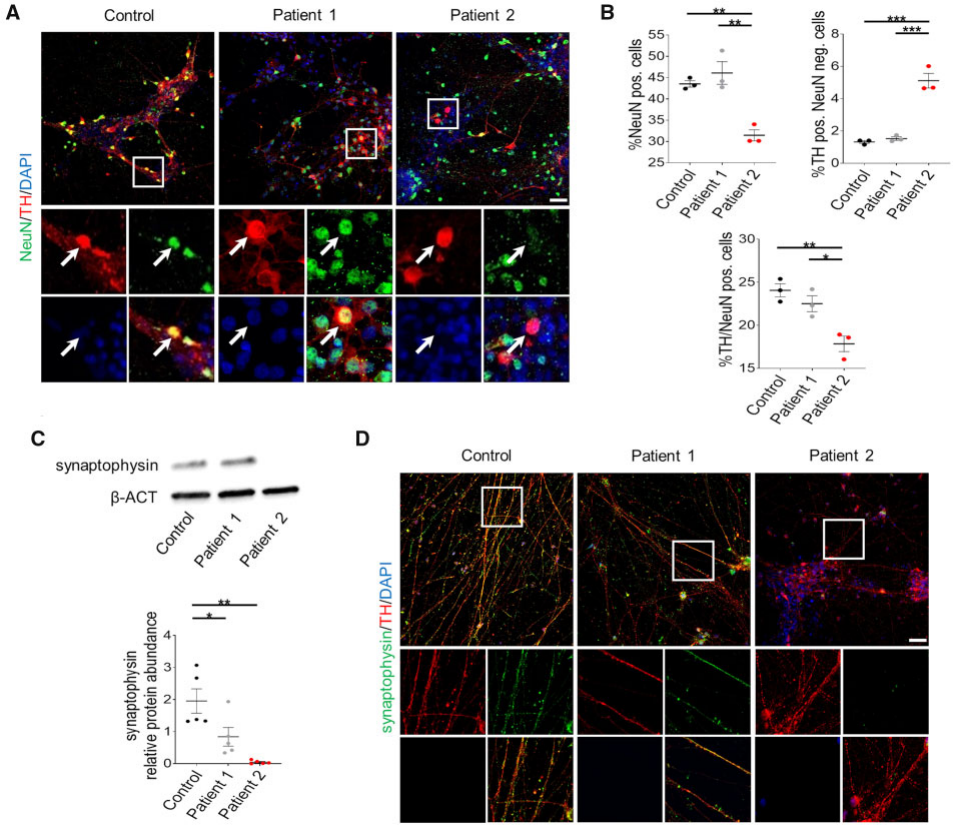
**Patient-derived neurons show defects in developmental maturation.** (**A**) Representative immunofluorescence images for NeuN and TH in control and patient-derived neurons. Arrows indicate double positive cells. Scale bar = 100 µm. *Insets* show higher magnification of NeuN-positive dopaminergic neurons. (**B**) Quantification of NeuN-positive, TH-positive and NeuN-negative, and TH/NeuN double-positive cells in derived neuronal cultures (*n* = 3 for all). (**C**) Representative immunoblot for synaptophysin and loading control (β-ACT) and quantification of relative synaptophysin abundance in total neuronal cell lysates (*n* = 5 for all). (**D**) Representative immunofluorescence for synaptophysin and TH in derived neurons. Scale bar = 100 µm. *Insets* show higher magnification of synaptophysin-positive dopaminergic neurons. Data are presented as mean ± SEM. *P < 0.05; **P < 0.01; ***P < 0.001, one-way ANOVA followed by Tukey’s multiple comparisons test.

We then sought to investigate the neurodevelopmental consequences of AADC deficiency in our *in vitro* model. Immunofluorescence analysis of the mature neuronal marker NeuN showed comparable levels in Patient 1 and control mDA neurons, while Patient 2 cultures showed a significant decrease in NeuN positivity when compared to both control and Patient 1 lines (Fig. 2A and B). Moreover, analysis of the vesicular protein synaptophysin revealed a significant decrease in protein levels for both Patients 1 and 2 when compared to control-derived neuronal cultures (Fig. 2C and D).

**Figure 3:**
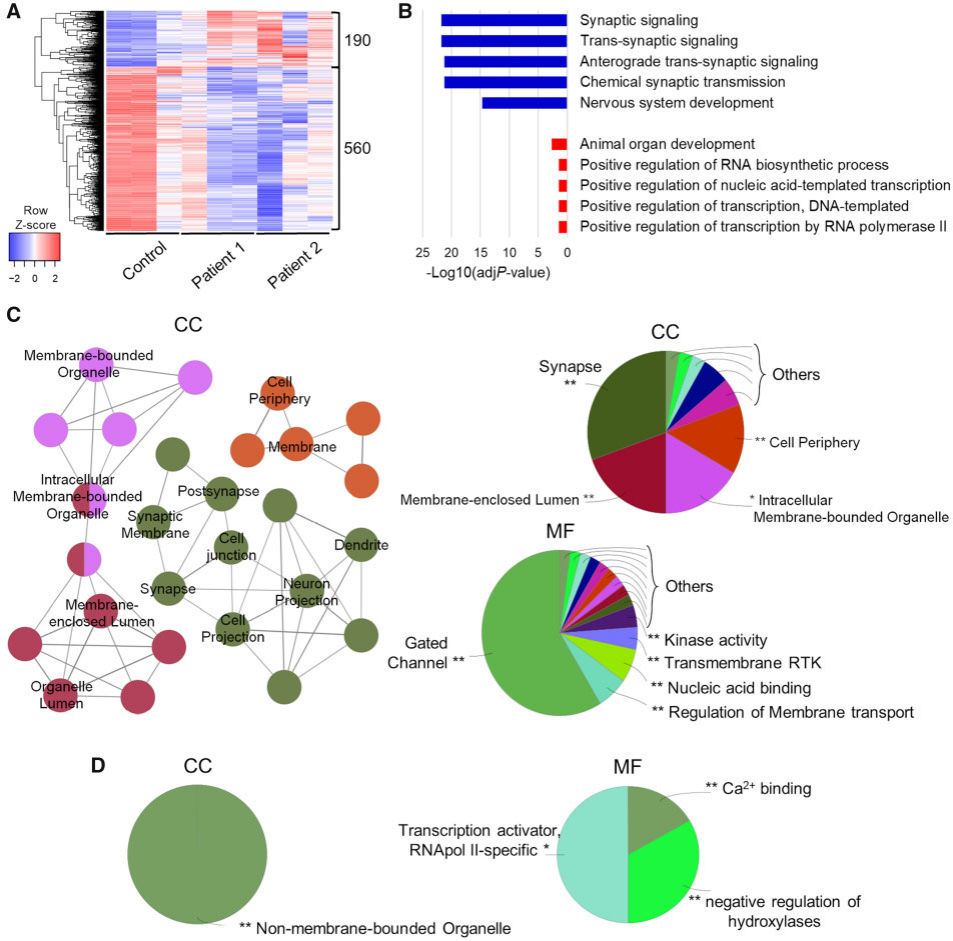
**Bulk RNA sequencing analysis shows an abnormal gene expression profile in AADC deficiency patients.** (**A**) Heat map showing hierarchical clustering of protein-coding DEGs in AADC deficiency patients compared to control (*n* = 3). (**B**) GO terms enrichment for biological process of underexpressed (blue) protein-coding and overexpressed (red) protein-coding DEGs. The top five categories are shown. (**C** and **D**) ClueGO analysis of GO terms enrichment of (**C**) under- and (**D**) over-expressed protein-coding DEGs, showing network graph and pie chart for cellular component (CC), and pie chart for molecular function (MF). Network graph nodes represent GO terms (the most significant are named) and edges indicate shared genes between GO terms. Functional groups of GO terms are indicated by the same colour. Pie charts show the percentages of each functional group representation, named with the most significant term. GO functional groups exhibiting higher statistically significant differences using Benjamini-Hochberg *P*-value correction (FDR < 0.05) are shown.

To investigate the neurodevelopmental effects of AADC deficiency, we undertook bulk RNA sequencing for analysis of DEGs between patient and control-derived neurons, with a particular focus on protein-coding genes. In a combined analysis of Patient 1 and Patient 2-derived neuronal cultures, we identified 750 DEGs (75% underexpressed and 25% overexpressed) when compared to the control (Fig. 3A). GO analysis of underexpressed DEGs revealed a strong enrichment in synaptic transmission-related biological processes and nervous system development, whilst overexpressed DEGs mainly enriched protein transcription and general organ developmental processes (Fig. 3B). Furthermore, underexpressed DEGs were associated with membranous cellular compartments (in particular, the cell periphery and synaptic region), and enriched in gated channels and regulators of membrane transport (Fig. 3C). In contrast, overexpressed DEGs were associated with non-membrane-bounded cell compartments (nucleus), with enrichment in transcriptional regulator proteins (Fig. 3D).

Considering the previously detected differences between the two patient lines (Fig. 2), single-comparison RNA sequencing analysis was also performed. We identified 842 protein-coding DEGs for Patient 1 compared to the control (Supplementary Fig. 6A) and 871 protein-coding DEGs for Patient 2 compared to the control (Supplementary Fig. 7A). For both analyses, underexpressed genes showed common enrichment for synaptic transmission (Supplementary Figs 6B and 7B)—reflected in the significant *P*-values observed in the combined analysis (Fig. 3B)—representing genes encoding proteins mainly localized at the cell periphery or synapses, and associated with ion channel function (Patients 1 and 2) and gated channel function (for Patient 2 in particular) (Supplementary Figs 6C and 7C). Differences in separate single Patient 1 and Patient 2 comparisons with the control were mainly detected for upregulated genes with regard to biological processes and significance (Supplementary Figs 6B and 7B): for Patient 1, overexpressed DEGs were enriched for developmental and cell projection assembly genes (Supplementary Fig. 6B and D), whereas for Patient 2 overexpressed DEGs were enriched for genes encoding endoplasmic reticulum and membrane-targeting processes and function (Supplementary Fig. 7B and D). Despite these inter-patient differences, the combined analysis reflects a common, disease-specific overexpression of developmental and transcriptional/translational processes from both single comparisons (Fig. 3B).

**Figure 4:**
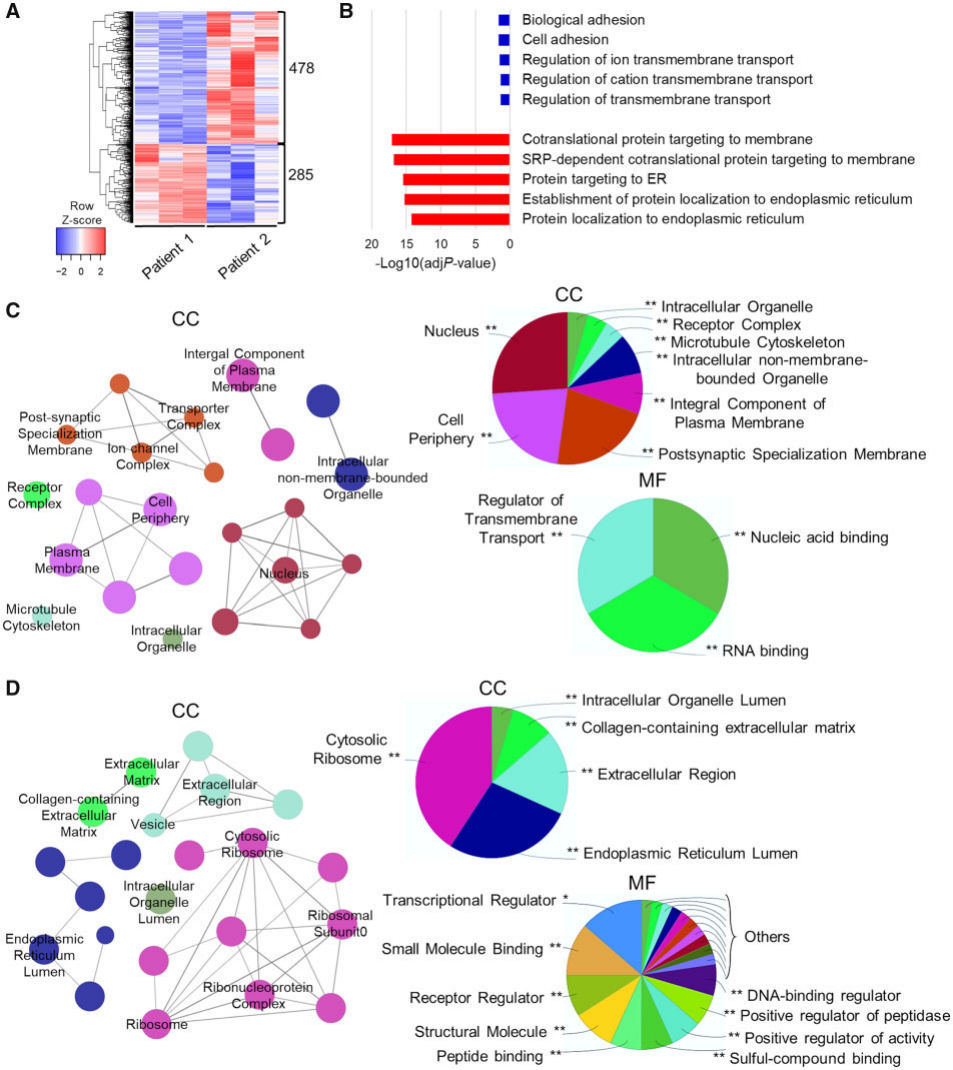
**Bulk RNA-Seq analysis reveals differences in gene expression profiles between Patient 1 and 2-derived neurons.** (**A**) Heat map showing hierarchical clustering of protein-coding DEGs in Patient 2, compared to Patient 1 (*n* = 3). (**B**) GO terms enrichment for biological process of underexpressed (blue) protein-coding and overexpressed (red) protein-coding DEGs. The top five categories are shown. (**C** and **D**) ClueGO analysis of GO terms enrichment of (**C**) under- and (**D**) over-expressed protein-coding DEGs, showing network graph and pie chart for cellular component (CC), and pie chart for molecular function (MF). Network graph nodes represent GO terms (the most significant are named) and edges indicate shared genes between GO terms. Functional groups of GO terms are indicated by the same colour. Pie charts show the percentages of each functional group representation, named with the most significant term. GO functional groups exhibiting higher statistically significant differences using Benjamini-Hochberg *P*-value correction (FDR < 0.05) are shown.

We then explored DEGs between the two different patient-derived neuronal cultures. We identified a total of 763 protein-coding DEGs for Patient 2 when compared to Patient 1 (Fig. 4A). The underexpressed DEGs showed enrichment in cell adhesion and membrane transport-related processes, while overexpressed DEGs enriched endoplasmic reticulum and membrane-targeting processes categories (Fig. 4B). Underexpressed DEGs corresponded to proteins localized both in cell periphery/membrane regions and nuclear compartment, with enrichment for genes regulating transcription and transmembrane transport (Fig. 4C). Overexpressed DEGs showed enrichment in both cytosolic transcriptional and extracellular compartments, with molecular functions mainly linked to structural/binding molecules, and transcriptional/activity regulators (Fig. 4D), resembling the result from the single comparison between Patient 2 and control (Supplementary Fig. 7B and D).

**Figure 5:**
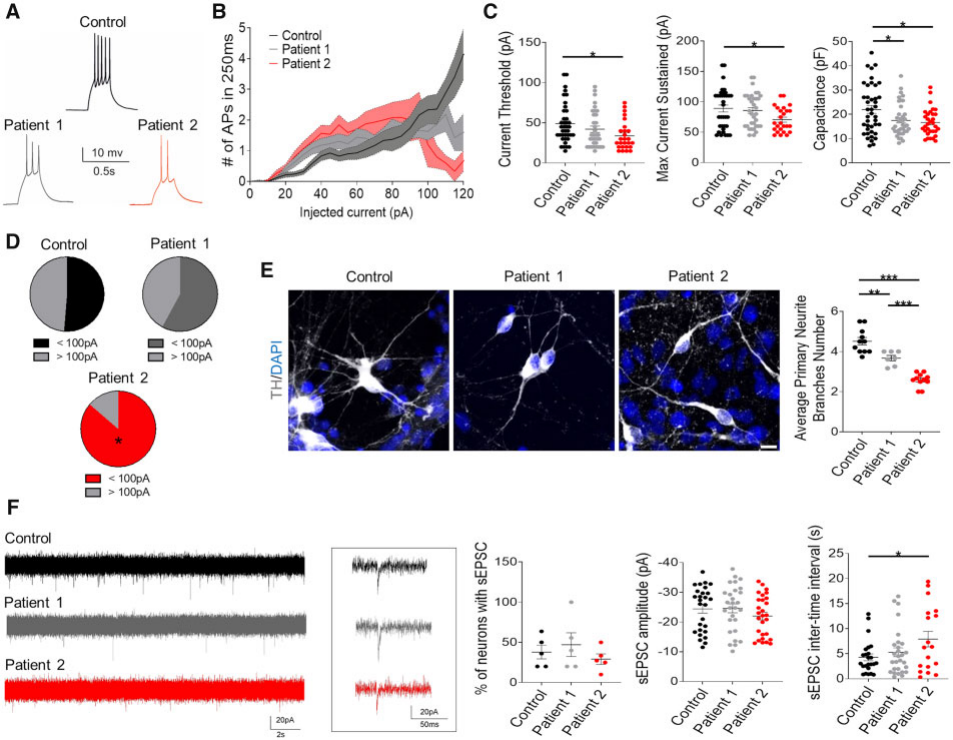
**Patient-derived neurons show altered neuronal electrophysiological properties and defects in primary neurite branching.** (**A**) Representative traces of action potentials (APs) elicited by injecting a 40 pA current in patients and control lines. (**B**) Input/output plot showing number of action potentials triggered by incremental current steps. (**C**) Active (current threshold and max current sustained) and passive (capacitance) properties of neurons in control, Patient 1 and Patient 2 neurons (*n* = 39, 34, 26, *n* = 35, 34, 25, and *n* = 41, 38, 32, respectively, from four biological replicates). (**D**) Percentage of neurons that sustain >100pA current injection. (**E**) Representative images for dopaminergic neurons branching (scale bar = 10 µm) and quantification of average primary neurite branching in control, Patient 1 and Patient 2 mDA neurons (*n* = 11, 7, 11, respectively). (**F**) Representative traces showing spontaneous excitatory postsynaptic currents (sEPSCs) at −70 mV and quantification of neurons with sEPSC, sEPSC amplitude and inter-time intervals in control, Patient 1 and Patient 2 neurons (*n* = 5 for all, *n* = 27, 28, 28, and *n* = 24, 28, 18, respectively, from four biological replicates). Data are presented as mean ± SEM. *P < 0.05; **P < 0.01; ***P < 0.001, one-way ANOVA followed by Tukey’s multiple comparisons test and chi-square test in **D.**

Whole-cell patch clamp electrophysiology studies were undertaken to determine whether the observed differences in gene expression were associated with functional differences in neuronal activity. The parameters analysed were similar to other studies using iPSC-derived dopaminergic neurons, with comparable findings for firing pattern, pacemaker and synaptic activity in controls.^34–37^ Recordings with increasing current amplitude (Fig. 5A) showed that the current threshold to elicit an action potential for Patient 2 was significantly lower than for the control (Fig. 5B and C) and failed to follow current injection up to 100 pA (Fig. 5D). On investigation of passive neuronal properties, both patients displayed lower capacitance compared to control neurons without affecting input resistance (Fig. 5C), in accordance with a decreased average number of primary neurite branches (Fig. 5E). For both control and patient-derived neurons showing spontaneous excitatory postsynaptic currents, we observed no differences in either the percentage of functionally connected neurons or current amplitude, although the interevent interval was significantly higher in Patient 2-derived neurons (Fig. 5F).

### *DDC* lentiviral gene transfer significantly improves neurodevelopmental defects in patient-derived neurons

**Figure 6:**
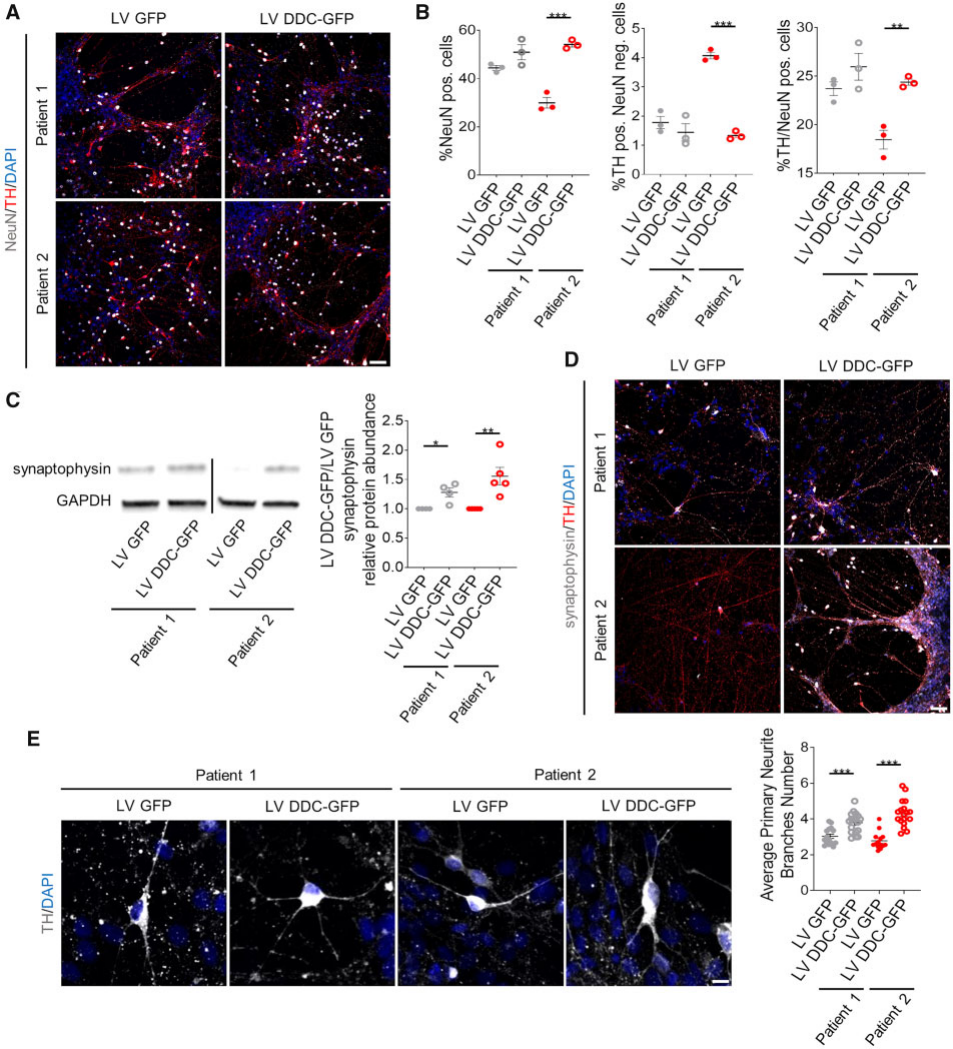
**Gene therapy significantly improves maturation defects in patient-derived neurons.** (**A**) Representative immunofluorescence for NeuN and TH of patient-derived neurons transduced with a lentiviral construct expressing only GFP (LV GFP) or human DDC and GFP (LV DDC-GFP). Scale bar = 100 µm. (**B**) Quantification of NeuN-positive, TH-positive and NeuN-negative, and TH/NeuN double-positive cells in patient-derived neuronal cultures transduced with LV GFP and LV DDC-GFP (*n* = 3 each). (**C**) Representative immunoblot for synaptophysin and loading control (GAPDH), and quantification of relative synaptophysin abundance from total cell lysates extracted from LV GFP and LV DDC-GFP transduced neurons. Results are normalized to the corresponding LV GFP for each patient (*n* = 4, 4, 5, 5, respectively). (**D**) Representative immunofluorescence for synaptophysin and TH in patient-derived neurons transduced with LV GFP or LV DDC-GFP. Scale bar =100 µm. (**E**) Representative images for dopaminergic neurons branching (scale bar = 10 µm) and quantification of average primary neurite branches in patient-derived neurons transduced with LV GFP or LV DDC-GFP (*n* = 15, 18, 13, 18, respectively). Data are presented as mean ± SEM. *P < 0.05; **P < 0.01; ***P < 0.001, two-tailed Student’s *t*-test.

Given that gene therapy is an emerging new treatment for AADC deficiency,^16–18^ we sought to investigate the cellular effects of human *DDC* transgene delivery in our model; in particular we wished to evaluate whether this therapeutic approach could improve the neurodevelopment sequelae of AADC deficiency, independent of genetic background. We generated a lentiviral construct for the delivery of human *DDC* under the control of the neuronal-specific promoter human synapsin (h*SYN1*) (Supplementary Fig. 8). Patient-derived mDA precursors were transduced at Day 24 of differentiation and analysed at Day 65. For both patient lines, lentiviral gene transfer resulted in an increase in AADC protein levels (Supplementary Fig. 9A and B), and rescued enzymatic activity to levels comparable to those observed in control neurons (Supplementary Fig. 9C). Furthermore, Patient 2 transduced neurons showed a significant increase in the NeuN-positive neuronal population, and in particular mDA neurons, to levels comparable to Patient 1 (Fig. 6A, B and Supplementary Fig. 10A). Human *DDC* lentiviral delivery also resulted in a significant increase in synaptophysin protein levels in both patients-derived neuronal cultures (Fig. 6C) and more specifically in the mDA neuronal subpopulation (Fig. 6D and Supplementary Fig. 10B), with a significant increase in primary branching (Fig. 6E).

### *In silico* and recombinant biochemical analyses predict a mutation-specific l-DOPA response in Patient 2

**Figure 7:**
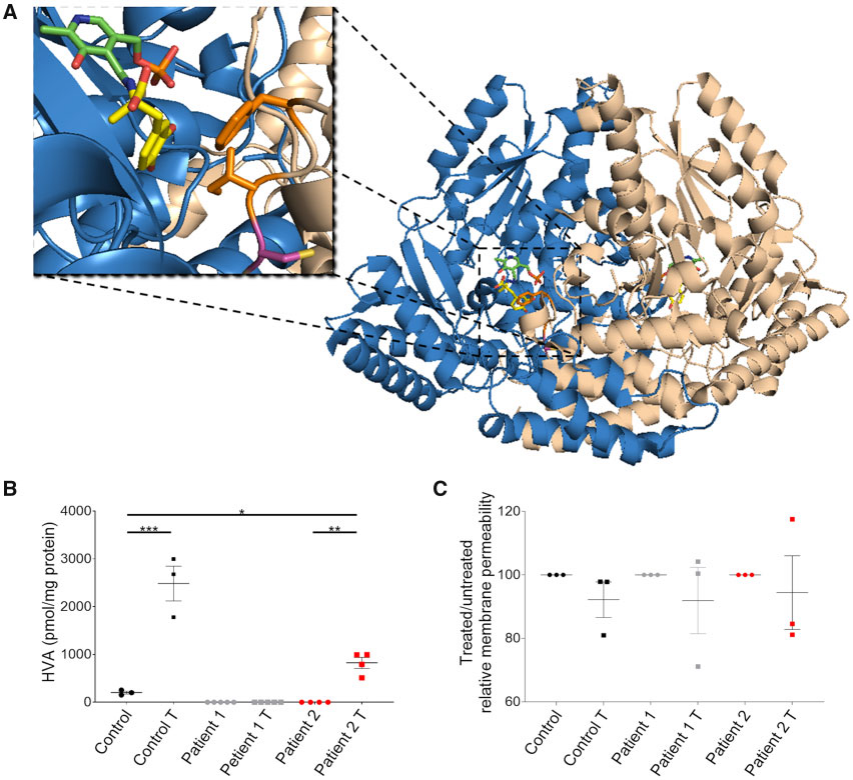
**l-DOPA treatment increases dopamine metabolite production in Patient 2-derived neuronal cultures, with no evidence of cellular toxicity.** (**A**) Localization of Cys100 in AADC protein structure. The structure corresponds to *Sus scrofa* holoenzyme (PDB code: 1JS3), solved in complex with PLP and carbidopa, and rendered using PyMol™ software. AADC is shown as a schematic, with the two monomers composing the native rearrangement of the enzyme (wheat and marine blue, respectively). PLP and carbidopa are represented as green and yellow sticks, respectively. The side chain of Cys100 is represented as a pink stick. Side chains of Ile101 and Phe103 are represented as orange sticks. (**B**) HPLC detection of extracellular HVA after 80 µM l-DOPA treatment of neuronal cultures for 24 h. Values are relative to total protein (*n* = 3, 3, 5, 5, 4, 4, respectively). (**C**) Dead-cell protease release assay after treatment. Results are normalized to the corresponding non-treated condition (*n* = 3 for all). Data are presented as mean ± SEM. *P < 0.05; **P < 0.01; ***P < 0.001, one-way ANOVA followed by Tukey’s multiple comparisons test.

The different mutations harboured by Patients 1 and 2 were further investigated to determine whether they had differential effects on enzymatic function. For Patient 1, despite supraphysiological levels of protein expression (Fig. 1C), the homozygous missense substitution R347G significantly impairs catalytic function of AADC leading to undetectable enzyme activity (Fig. 1A) without impacting the protein structure by a molecular mechanism extensively investigated in Montioli *et al*.^15^ In contrast, despite significantly low levels of AADC protein in Patient 2-derived neuronal cultures (Fig. 1C), residual enzymatic activity was still detected, albeit at a fraction of that evident in the control line (Fig. 1A). It is likely that this residual AADC enzyme activity can be attributed to the p.C100S variant, since the second heterozygous mutation leads to an early stop codon at Arg7, predicted to result in nonsense-mediated mRNA decay and absent protein production. The missense mutation C100S results in an amino acid substitution at the beginning of an essential loop (loop 2, residues 100–110), which contains key hydrophobic active site residues involved in substrate binding, in particular Ile101 and Phe103.^38^ The cysteine-to-serine amino acid substitution has the potential to alter the conformation of loop 2 and consequently the substrate-binding cleft, thereby affecting substrate affinity (Fig. 7A). AADCC100S was produced *in vitro* in recombinant form to characterize the effects of this mutation through spectroscopic, circular dichroism and fluorescence analyses, and calculation of kinetic parameters. A minor perturbation of PLP cofactor microenvironment (in particular for the enolimine tautomer) was observed (Supplementary Fig. 11A and B). However, PLP binding affinity (Supplementary Table 1) was not particularly affected, with a K_D(PLP)_ consistently lower than previously reported values for AADC variants with cofactor binding impairment.^13^ Calculation of kinetic parameters (Supplementary Table 1) revealed that AADCC100S retains more residual enzyme activity than that reported for other AADC variants.^12^^,^^13^ The catalytic activity (*k_cat_*) of AADCC100S was indeed similar to that observed for wild-type, with the actual decrease in overall AADCC100S catalytic efficiency (*k_cat_*/K_M_) attributed to a slight decrease in l-DOPA affinity (K_M_) (Supplementary Table 1). As such, we postulated that dopamine production by AADCC100S could be enhanced with l-DOPA administration, as demonstrated for other AADC variants.^39^

### Patient 2-derived midbrain dopaminergic neurons specifically respond to l-DOPA administration

To determine whether the C100S mutation resulted in l-DOPA responsivity, we sought to investigate the effect of l-DOPA treatment in Patient 2-derived mDA neurons. After 65 days of differentiation, both patients and control-derived neuronal cultures were incubated with 80 µM l-DOPA for 24 h, a dose just below that considered to be toxic in neuronal and other cellular systems.^40^^,^^41^ Subsequent HPLC analysis of extracellular metabolites was then undertaken. As expected in a system with catalytically competent AADC, HVA levels were significantly higher in treated control compared to untreated control neurons (Fig. 7B). Furthermore, as predicted, there was no detectable HVA in Patient 1-derived neuronal cultures both pre and post l-DOPA treatment. However, for Patient 2, we observed a significant increase of HVA levels in l-DOPA treated cultures when compared to untreated cultures (Fig. 7B). To evaluate any potential toxicity related to l-DOPA administration^40^ or dopamine production,^42^ we measured dead-cell protease release and found no increase in membrane permeability for both patients and control-derived neuronal cultures treated with 80 µM l-DOPA for 24 h (Fig. 7C). Moreover, analysis of JNK protein phosphorylation, which increases in response to toxic levels of dopamine,^43^ showed a significant increase in the phosphorylated form of this kinase in treated control neurons only, while no significant increase was detected in both treated Patient 1 and 2 cultures (Supplementary Fig. 11C).

## Discussion

AADC deficiency is a complex and often pharmacoresistant neurological disorder, with a broad phenotypic spectrum, variable drug response, substantial burden of disease and significant risk of premature mortality.^7^ Improved understanding of the underlying pathogenic mechanisms and the development of better targeted treatments, such as gene therapy and other personalized medicine approaches, will be key in modifying disease and long-term outcome. In this study, we have developed a new humanized model of AADC deficiency. Our *in vitro* patient-derived mDA neuronal model of AADC deficiency has provided further insight into mechanisms governing disease, as well as an ideal system to evaluate the impact of approaches such as gene therapy at cellular level and a unique research platform to evaluate mutation-specific precision medicine approaches.

Importantly, our patient-derived mDA model recapitulates key features of the human phenotype with near-absent AADC enzyme activity and impaired dopamine metabolism. In our dopaminergic model, we observed a greater degree of residual AADC enzyme activity in Patient 2, which may relate to the more advanced motor gains observed in this patient. We also observed patient-specific altered levels of AADC protein. The reasons for this are not entirely clear, given that little is known about factors that govern AADC enzyme regulation. Our biochemical investigations did not show a differential intrinsic protein stability between mutant and wild-type protein. We did, however, observe a clear patient-specific increase in *DDC* gene expression for Patient 1 and higher than expected levels of *DDC* expression for Patient 2*,* given the predicted nonsense-mediated decay of a proportion of Patient 2 transcripts. Furthermore for both patients there was an increase in TH gene and protein expression; interestingly, TH gene and protein expression has previously been shown to increase in Parkinson’s disease, as a likely compensatory response to a state of dopamine deficiency in the context of striatonigral degeneration.^44^^,^^45^ As such, it is plausible that the similar state of dopamine deficiency in AADC-deficient patient lines drives a positive feedback mechanism to modulate neuronal levels of key enzymes driving dopamine synthesis.

Moreover, our study suggests that AADC dysfunction may have widespread effects on gene expression that may impact neuronal development and functional maturation. As well as its pivotal role in monoamine neurotransmission, dopamine is postulated to have important functions in modulating neuronal structure and connectivity.^46^ The early production of dopamine in midbrain development suggests that it may have neurodevelopmental influence,^47^ a notion that is further corroborated by *DDC* knock-in mice and knockout zebrafish which show abnormal development.^48^^,^^49^ Interestingly, our patient-derived cell model also shows that defective AADC enzymatic activity and dysregulated dopamine metabolism affects neuronal maturity, with altered expression of genes involved in neurodevelopment and synaptic formation, as well as disruption of electrophysiological properties and functional activity. Considering that iPSC-derived neurons resemble foetal neurons,^50^ it is possible that the neuronal maturation defects observed in our *in vitro* model correlate with prenatal disease onset in humans. This is not surprising, given that many affected patients present with their first clinically discernible symptoms in early infancy. Our results are particularly relevant in the current climate of emerging gene therapy approaches,^18^ where neuronal plasticity is considered to be an important requisite for clinical benefit.^51^ It is likely that gene therapy within this ‘therapeutic window’ of brain plasticity may predict a more favourable long-term neurodevelopmental outcome.

In our system, we identified around the same number of differentially expressed genes between patients (when combined) and control and between the two patients; the latter observation likely reflects both the biological and clinical differences between patients affected by a disease with a broad phenotypic continuum. Patient 2-derived cultures showed indeed a greater degree of neuronal immaturity. Notably, Patient 2 had a number of behavioural issues, significant autistic traits and prominent neuropsychiatric symptoms, features that were less evident in Patient 1. Our data may indicate that the greater degree of neuronal immaturity evident in Patient 2 lines as seen on maturation marker analysis, transcriptome profiling and electrophysiology may contribute to the aforementioned neurodevelopmental symptoms. Importantly, lentiviral treatment of patient-derived neurons restored AADC protein levels and enzymatic activity with significant improvement in neuronal maturity. Whether AADC protein has additional functions in governing neurodevelopment processes, that are independent of its catalytic activity in dopamine production, remains yet to be determined. Further studies with a greater number of patient lines and age-matched/isogenic controls, or analysis of multiple clones from each line, will help confirm and further delineate the complex neurodevelopmental biological phenotypes identified in this study. Additional genetic, epigenetic and environmental factors may also play a role in such phenotypic variability seen in the cell model and human phenotype; over time, advances in next generation sequencing technologies may also help further elucidate some of the underlying contributory genetic factors.

Our patient-derived model of AADC deficiency has proven to be a useful tool for evaluating therapeutic approaches. We have shown recovery of AADC enzyme activity and specific neuronal maturation defects using a gene therapy approach in patient-derived neurons. In tandem with other models, such therapeutic testing in iPSC-based systems may in the future guide and influence clinical trial design. Our model has also demonstrated the potential utility of l-DOPA treatment for some patients with AADC deficiency. Although l-DOPA is not traditionally used in the majority of patients,^8^ it has been previously empirically used in patients suspected to have l-DOPA responsive AADC deficiency.^39^ Our study confirms that it may indeed have a role for patients with specific *DDC* mutations associated with residual enzymatic activity due to altered substrate affinity. A planned therapeutic trial will further inform whether the positive effects of l-DOPA observed *in vitro* are recapitulated *in vivo.* More generally, our study shows that better definition of the physiochemical properties of specific mutations with subsequent validation in patient-relevant models has great potential in guiding personalized pharmacological strategies for rare disorders.

In conclusion, as new therapeutic avenues emerge for patients with AADC deficiency, our study shows the clear utility of an iPSC-based modelling system to elucidate disease mechanisms and evaluate therapeutic strategies.

## Supplementary Material

awab123_Supplementary_Data

## Abbreviations

AADC: aromatic l-amino acid decarboxylase
DEGs: differentially expressed genes
HPLC: high performance liquid chromatography
HVA: homovanillic acid
iPSC: induced pluripotent stem cell
l-DOPA: l-3, 4-dihydroxyphenylalanine
mDA: midbrain dopaminergic
PLP: pyridoxal 5′-phosphate
