# Supplementary material for: Aromatic l-amino acid decarboxylase deficiency: a patient-derived neuronal model for precision therapies
Source: Brain. 2021 Mar 18;144(8):2443–56. doi: 10.1093/brain/awab123 (PMC8418346; doi:10.1093/brain/awab123)
Supplement: awab123_Supplementary_Data [file awab123_supplementary_data.zip › awab123-suppl_data/OP-BRAI210122_PECorr_CmtAttachmentsFolder_Supplementary Data_Figures and Tables.pdf]

Aromatic L-Amino Acid Decarboxylase Deficiency: A Patient-derived Neuronal Model for Precision Therapies

Giada Rossignoli[1,2], Karolin Krämer[1], Eleonora Lugarà[3], Haya Alrashidi[4], Simon Pope[5], Carmen De La Fuente Barrigon[4], Katy Barwick[1], Giovanni Bisello[2], Joanne Ng[1,6], John Counsell[1], Gabriele Lignani[3], Simon J. R. Heales[5,7], Mariarita Bertoldi[2,*], Serena Barral[1], Manju A. Kurian[1,8,*]

Affiliations

1. Developmental Neurosciences, GOS Institute of Child Health, University College London, London WC1N 1EH, UK
2. Biological Chemistry, NBM Department, University of Verona, 37134 Verona, Italy
3. Clinical and Experimental Epilepsy, Queen Square Institute of Neurology, University College London, London WC1N 3BG, UK
4. Genetics and Genomic Medicine, GOS Institute of Child Health, University College London, London WC1N 1EH, UK
5. Neurometabolic Unit, National Hospital for Neurology and Neurosurgery, Queen Square, London WC1N 3BG, UK
6. Gene Transfer Technology Group, EGA-Institute for Women's Health, University College London, London WC1E 6HU, UK
7. Centre for Inborn Errors of Metabolism, GOS Institute of Child Health, University College London, London WC1N 1EH, UK
8. Department of Neurology, Great Ormond Street Hospital, London WC1N 3JH, UK

*Correspondence to:

Prof Manju Kurian; Zayed Centre for Research, UCL Great Ormond Street Institute of Child Health, 20 Guilford St, London WC1N 1DZ, UK. Email: manju.kurian@ucl.ac.uk

Prof Mariarita Bertoldi; Department of Neuroscience, Biomedicine and Movement Sciences, Biological Chemistry Section, Room 1.24, Strada le Grazie 8, 37134 Verona, Italy. E-mail: mita.bertoldi@univr.it

Supplementary Figures:

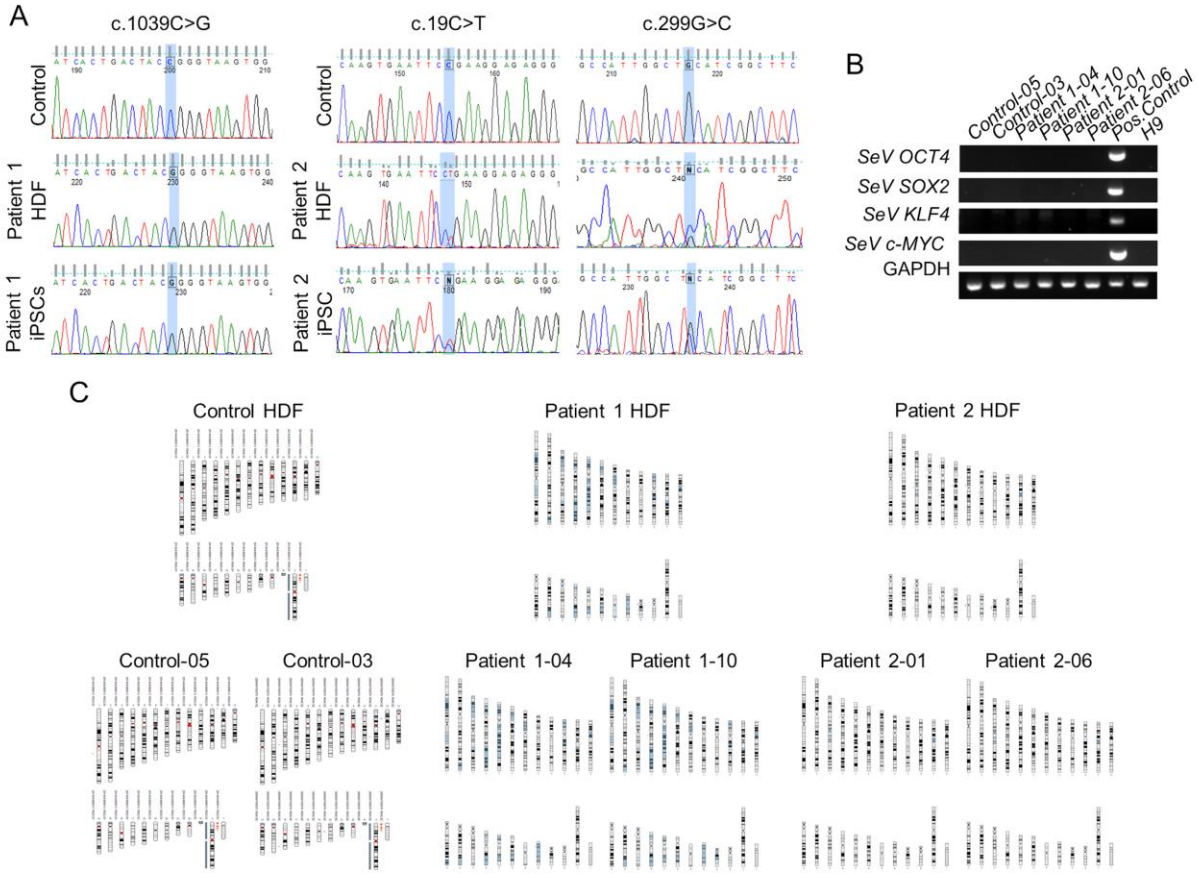

Supplementary Fig. 1: Control and patients-derived iPSC lines retain genomic integrity. (**A**) Sequence chromatograms of Control and Patients human dermal fibroblasts (HDF), and derived-iPSCs showing retention of the *DDC* mutations post reprogramming (blue boxes). (**B**) RT-PCR for exogenous Sendai Virus genes (SeV) *OCT4*, *SOX2*, *KLF4*, *c-MYC* in derived iPSCs, positive control and human Embryonic Stem Cells (H9). (**C**) Illumina Infinium HumanCytoSNP-12 v2.1 BeadChip array analysis for original Control and Patients HDFs, and derived iPSC lines (Control-05, Control-03; Patient 1-04, Patient 1-10; Patient 2-01, Patient 2-06).

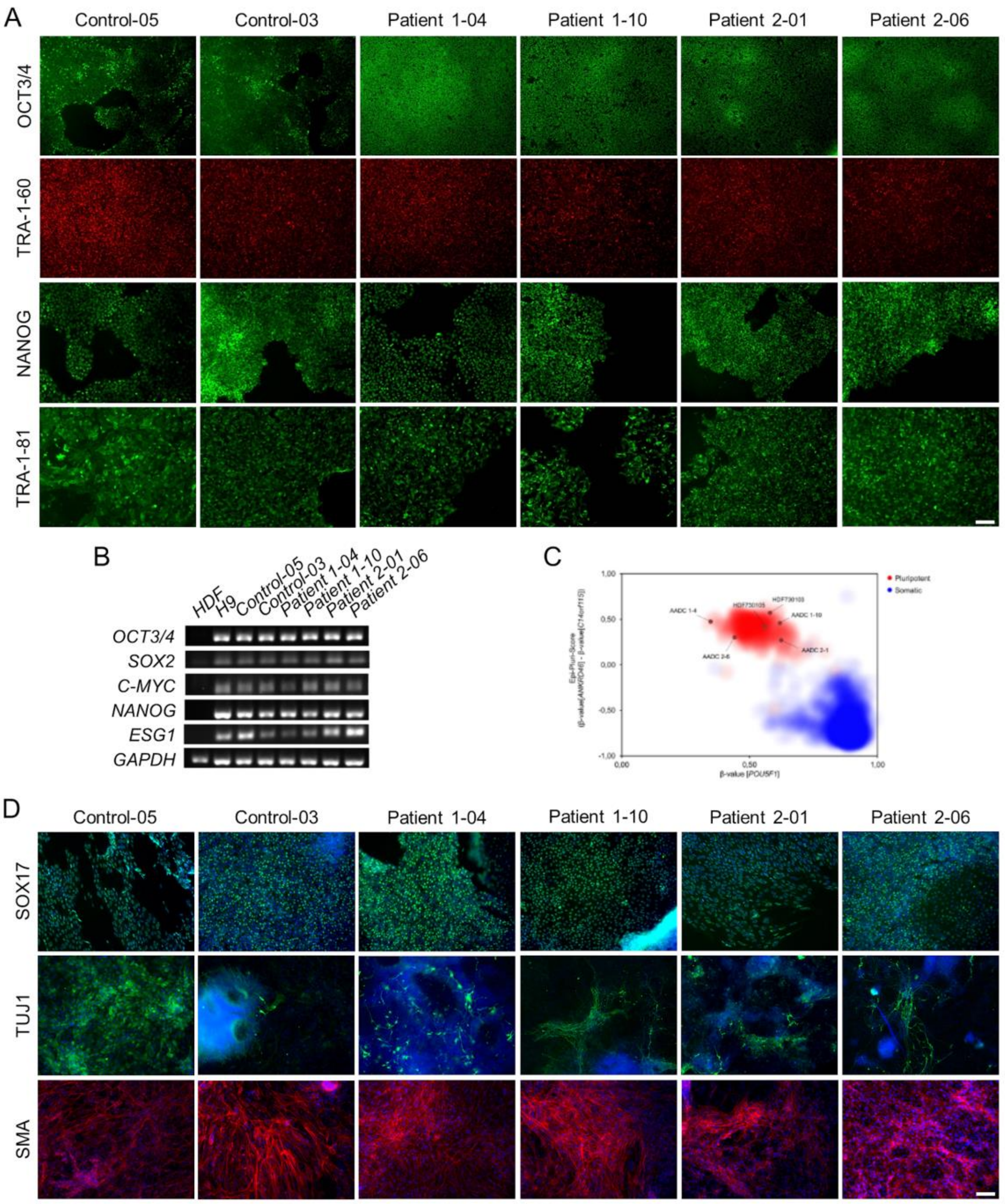

Supplementary Fig. 2: Control and patient-derived iPSC lines show true pluripotency.
(**A**) Immunofluorescence analysis for OCT3/4, TRA-1-60, NANOG, and TRA-1-81 in Control and Patients-derived iPSC lines. Scale bar 200 µm. (**B**) RT-PCR for pluripotency genes *OCT3/4*, *SOX2*, *C-MYC*, *NANOG* and *ESG1* expression in HDF, human Embryonic Stem Cells (H9) and derived iPSC lines. (**C**) Epi-Pluri-Score analysis for pluripotency (Illumina HumanMethylation27 BeadChip platform) for Control and Patents-derived iPSC lines, based on DNA-methylation profiles of 264 pluripotent (Red cloud) and 1,951 non-pluripotent (blue cloud) cell preparations. (**D**) Immunofluorescence representative images for SOX17 (endoderm), TUJ1 (ectoderm) and SMA (mesoderm) expression from spontaneous *in vitro* differentiation of Control and Patient-derived iPSC lines. Scale bar 200 µm.

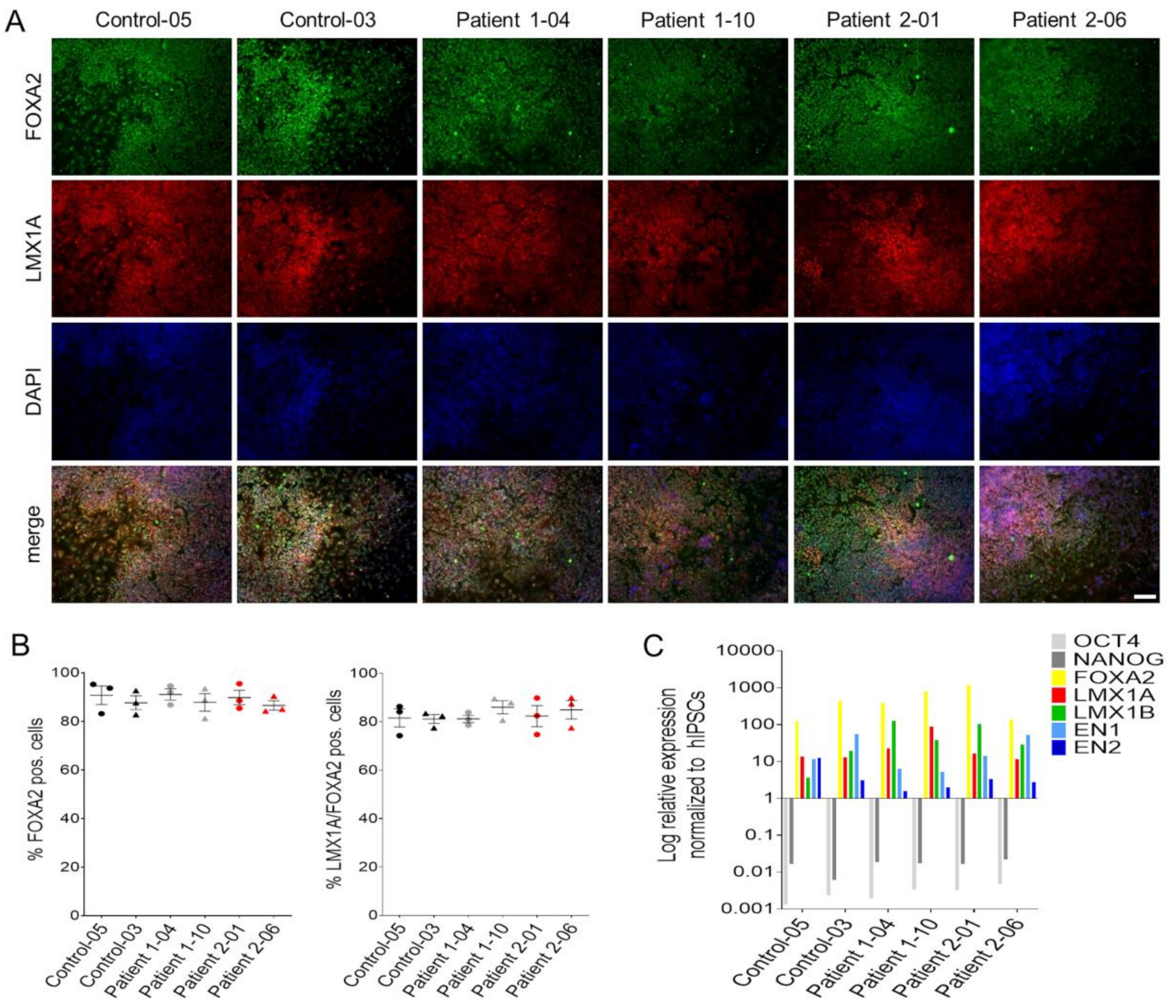

Supplementary Fig. 3: Control and patient-derived iPSC lines differentiate efficiently into mDA precursors.

(**A**) Representative immunofluorescence images for FOXA2 and LMX1A in Control and Patient-derived progenitors after 11 days of differentiation. Scale bar 200µm. (**B**) Quantification of total number of FOXA2-positive cells and LMX1A/FOXA2 double-positive cells at Day 11 (n=3 for all). (**C**) Representative qRT-PCR at Day 11 of *OCT4* and *NANOG* for pluripotency, and *FOXA2*, *LMX1A*, *LMX1B*, *EN1* and *EN2* for midbrain specification, relative to housekeeping gene (GAPDH) and normalized to their respective iPSC lines. Data are represented as mean ±SEM. *P < 0.05; **P < 0.01; ***P < 0.001, one-way ANOVA followed by Tukey's multiple comparisons test.

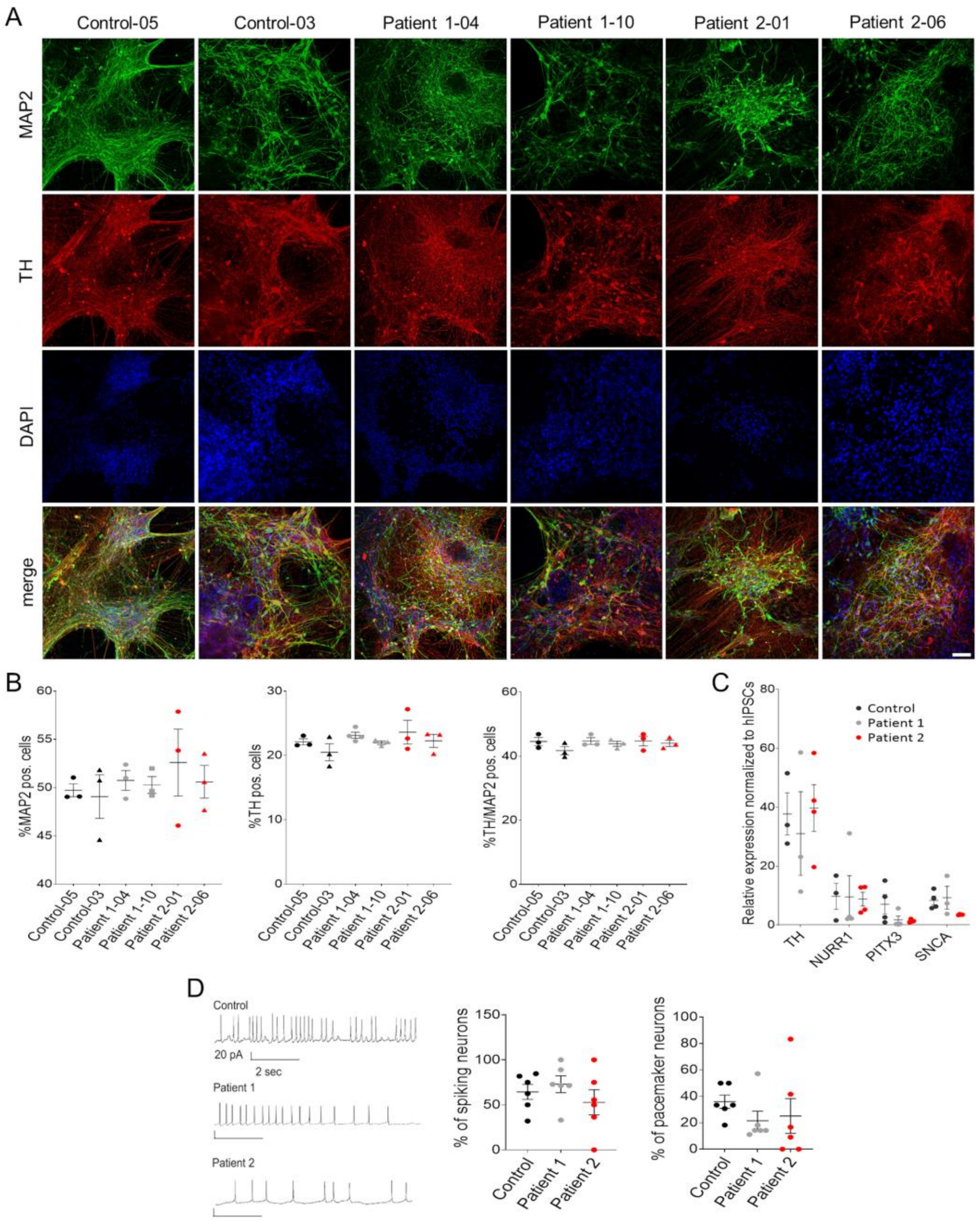

Supplementary Fig. 4: Control and patient-derived iPSC lines differentiate into mature mDA neurons.

(**A**) Representative immunofluorescence images for MAP2 and TH of derived neurons at Day 65. Scale bar 150µm. (**B**) Quantification of total number of MAP2 and TH positive cells, and TH/MAP2 double-positive cells at Day 65 (n=3 for all). (**C**) qRT-PCR analyses for *TH*, *NURR1*, *PITX3* and *SNCA* at Day 65 of maturation. mRNA levels are relative to the housekeeping gene (GAPDH) and normalized to the corresponding iPSC line (n≥3 for all). (**D**) Representative traces of multiple APs elicited by current injection (60pA), and quantification of the total number of spiking neurons and pacemaker spiking neurons patched for electrophysiology analysis at Day 65 (n=6 for all). Data are represented as mean ±SEM. *P < 0.05; **P < 0.01; ***P < 0.001, one-way ANOVA followed by Tukey's multiple comparisons test.

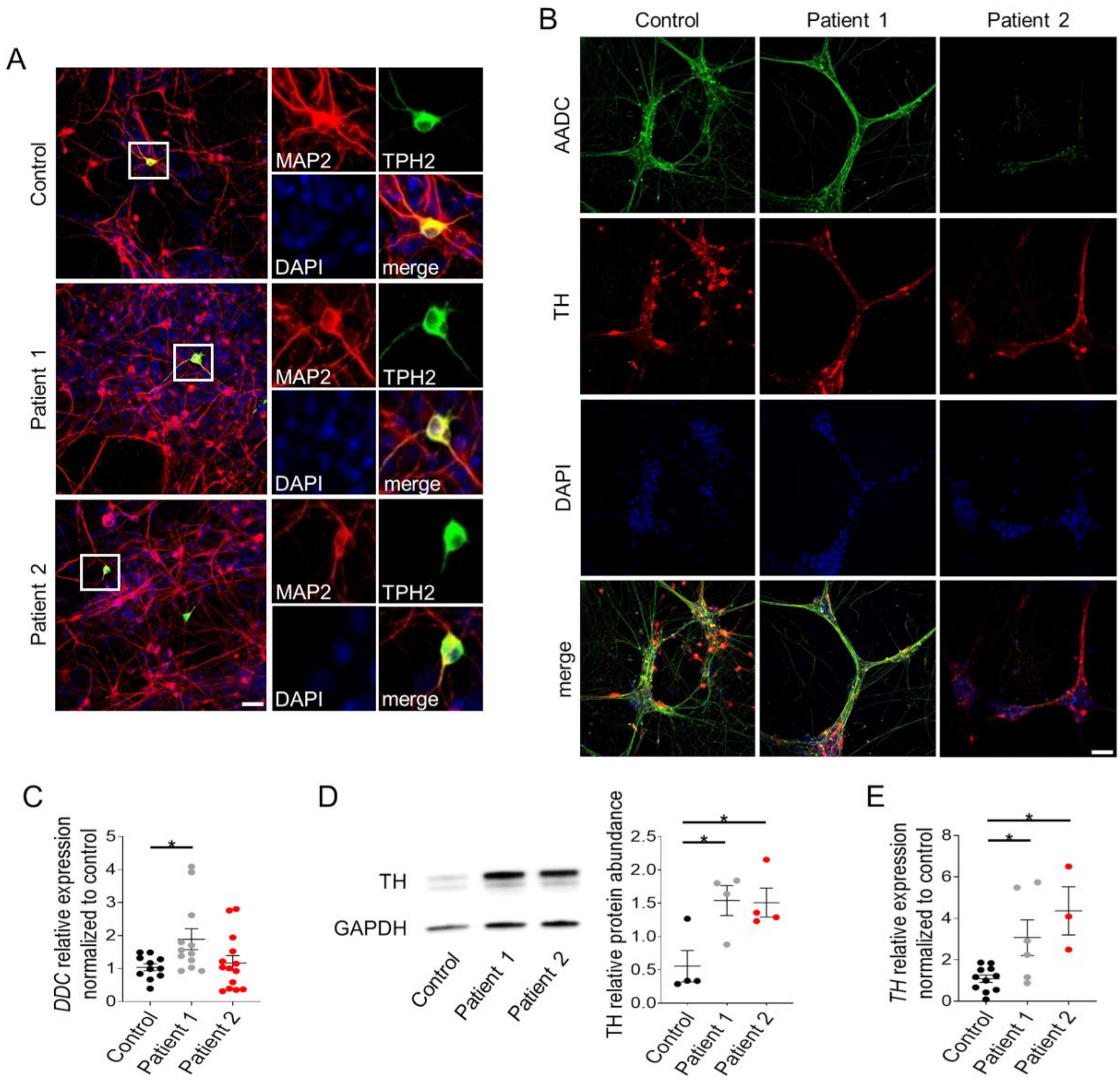

Supplementary Fig. 5: Patient-derived neurons show dysregulated dopaminergic pathway.

(**A**) Representative immunofluorescence image of TPH2 and MAP2 in derived neuronal cultures. Scale bar 100µm. Inserts show higher magnification of TPH2-positive neurons. (**B**) Representative immunofluorescence images for AADC in derived neurons. Cells are stained for TH/AADC, complete panel of Fig. 1D. Scale bar 150µm. (**C**) qRT-PCR analysis for *DDC* at Day 65 of maturation. mRNA levels are relative to the housekeeping gene (GAPDH) and normalized to the Control (n=11, 12, 14, respectively). (**D**) Immunoblot analysis for TH protein in Control, Patient 1 and Patient 2 derived neurons at Day 65 of differentiation. Quantification relative to loading control (GAPDH) (n=4 for all). (**E**) qRT-PCR analysis for *TH* at Day 65 of maturation. mRNA levels are relative to the housekeeping gene (GAPDH) and normalized to the Control (n=11, 6, 3, respectively). Data are represented as mean ±SEM. *P < 0.05; **P < 0.01; ***P < 0.001, one-way ANOVA followed by Tukey's multiple comparisons test

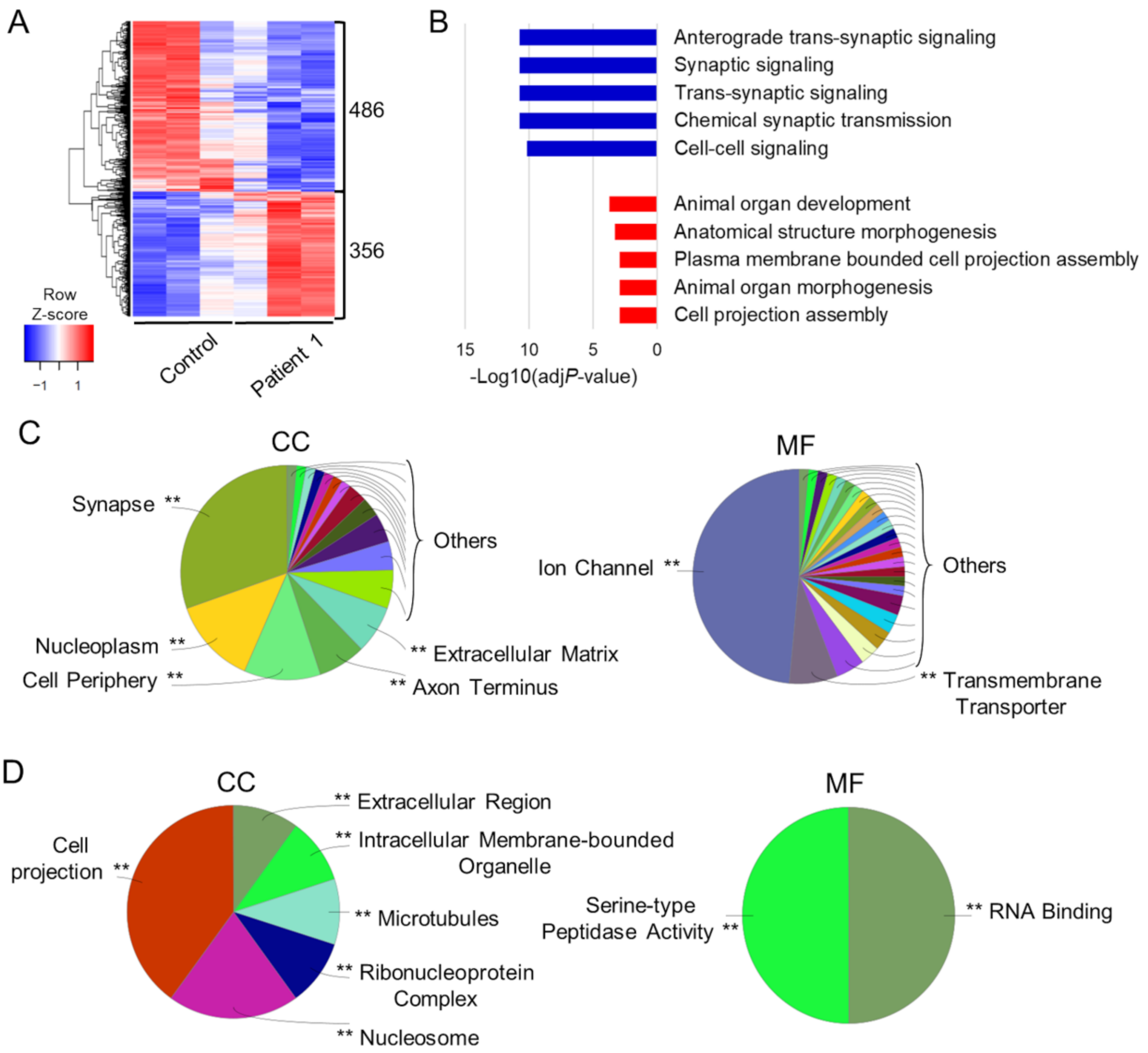

Supplementary Fig. 6: Bulk RNA-Seq analysis of Patient 1 and Control derived-neurons. (**A**) Heat map showing hierarchical clustering of protein-coding DEGs in Patient 1 compared to Control (n=3). (**B**) GO terms enrichment for biological process of underexpressed (blue) protein-coding and overexpressed (red) protein-coding DEGs. The top five categories are shown. (**C**-**D**) ClueGO analysis of GO terms enrichment of under- (**C**) and over- (**D**) expressed protein-coding DEGs, showing pie charts for cellular component (CC) and molecular function (MF). Pie charts show the percentages of each functional group representation, named with the most significant term. GO functional groups exhibiting higher statistically significant differences using Benjamini-Hochberg P-value correction (FDR < 0.05) are shown.

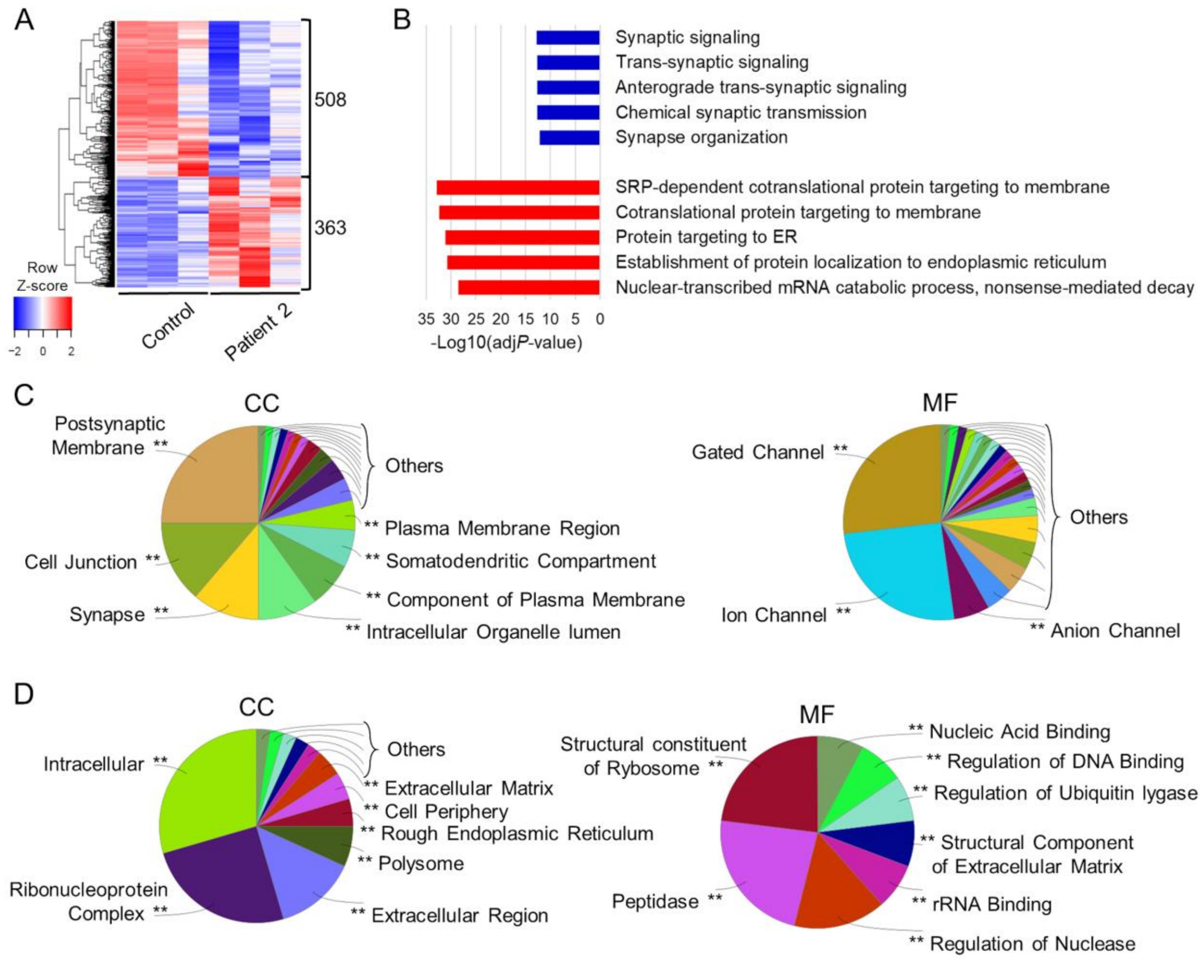

Supplementary Fig. 7: Bulk RNA-Seq analysis of Patient 2 and Control derived-neurons. (**A**) Heat map showing hierarchical clustering of protein-coding DEGs in Patient 2 compared to Control (n=3). (**B**) GO terms enrichment for biological process of underexpressed (blue) protein-coding and overexpressed (red) protein-coding DEGs. The top five categories are shown. (**C**-**D**) ClueGO analysis of GO terms enrichment of under- (**C**) and over- (**D**) expressed protein-coding DEGs, showing pie charts for cellular component (CC) and molecular function (MF). Pie charts show the percentages of each functional group representation, named with the most significant term. GO functional groups exhibiting higher statistically significant differences using Benjamini-Hochberg P-value correction (FDR < 0.05) are shown.

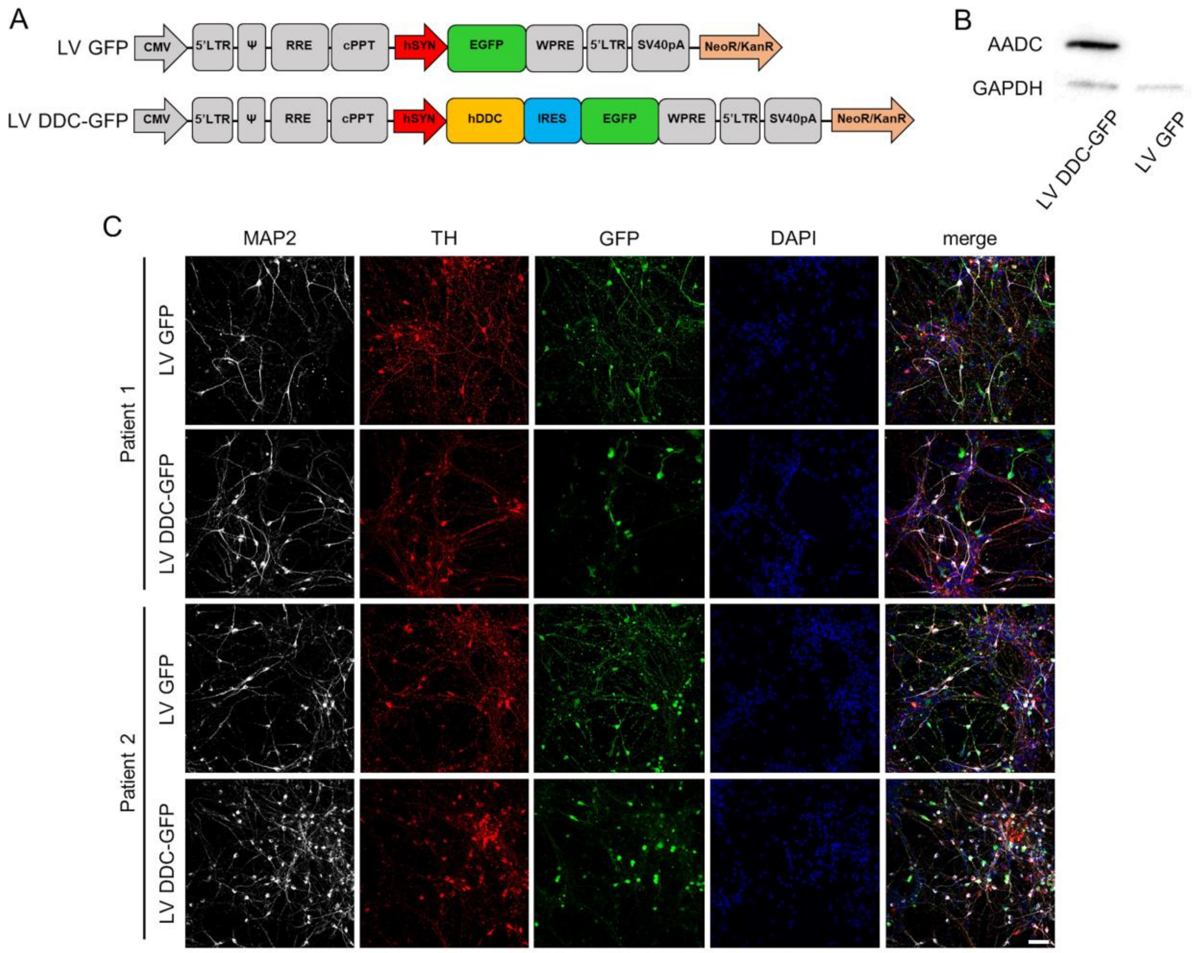

Supplementary Fig. 8: Generation of a lentiviral construct for *in vitro* gene transfer.

(**A**) Schematic representation of the lentiviral construct composed of an expression cassette with human synapsin promoter (h*SYN*1) driving human *DDC* gene (h*DDC*) linked by Internal Ribosome Entry Site sequence (IRES) to GFP gene. (**B**) Representative immunoblot for AADC and loading control (GAPDH) from total lysate of HEK 293T cells 72 h after transfection with LV GFP and LV DDC-GFP. (**C**) Representative immunofluorescence images of patient-derived neuronal cultures transfected with LV GFP and LV DDC-GFP at Day 24 and stained for MAP2/TH/GFP at Day 65 of maturation. Scale bar 150µm.

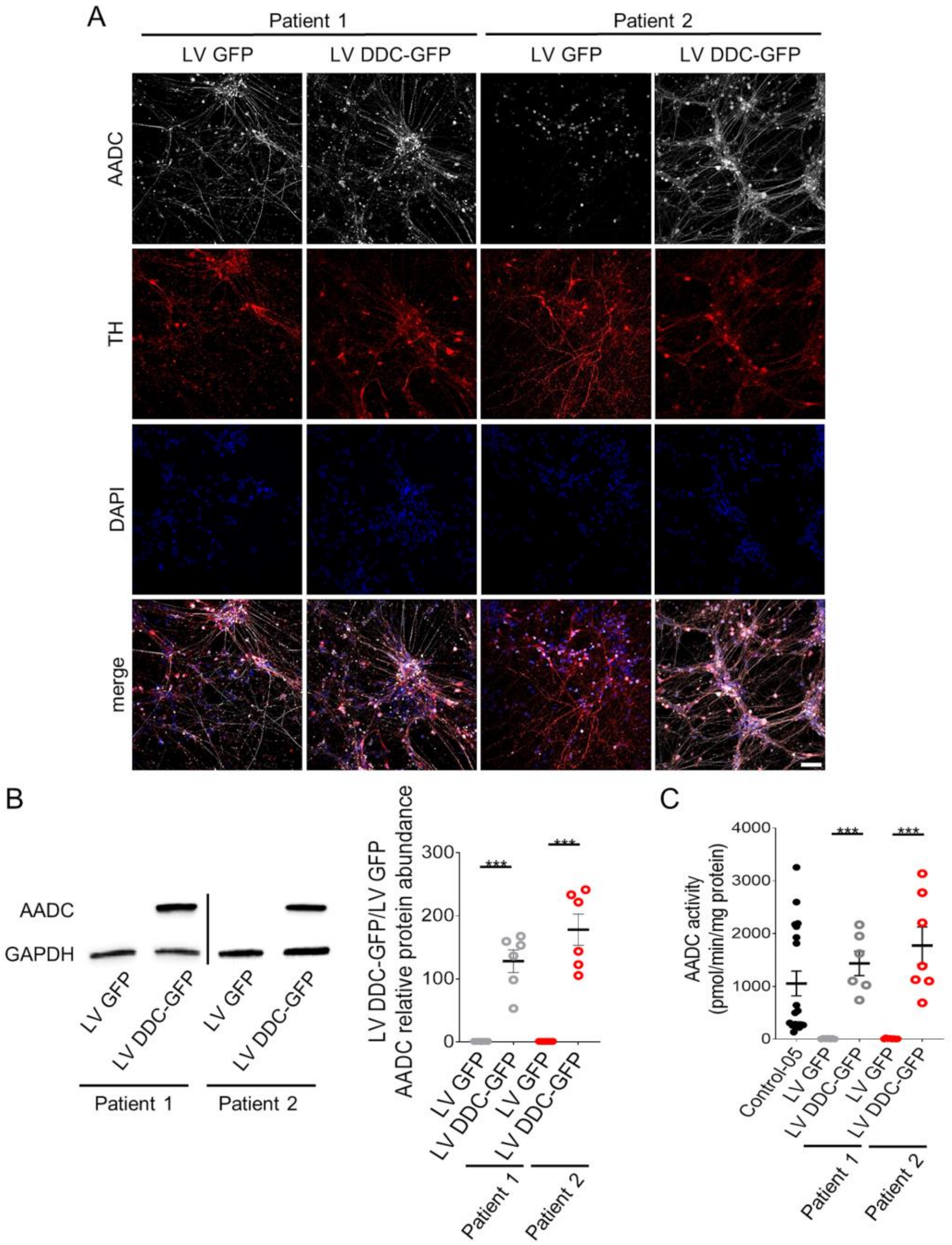

Supplementary Fig. 9: Gene therapy treatment of patient-derived neurons restores AADC protein and enzymatic activity.

(**A**) Representative immunofluorescence for AADC expression in patient-derived neurons transduced with LV GFP and LV DDC-GFP. Scale bar 100µm. (**B**) Representative immunoblot for AADC and loading control (GAPDH) and quantification of relative AADC abundance from total proteins extracted from LV GFP and LV DDC-GFP transduced patient-derived neuronal cultures. Results are normalized to LV GFP levels for each patient (n=6 for all). (**C**) AADC activity assay for untreated Control, and Patient 1/Patient 2 neurons transduced with LV GFP and LV DDC-GFP. Values are relative to total protein (n=19, 7, 6, 8, 7 respectively). Data are represented as mean ±SEM. $^{*}P < 0.05$; $^{**}P < 0.01$; $^{***}P < 0.001$, two-tailed Student's *t*-test.

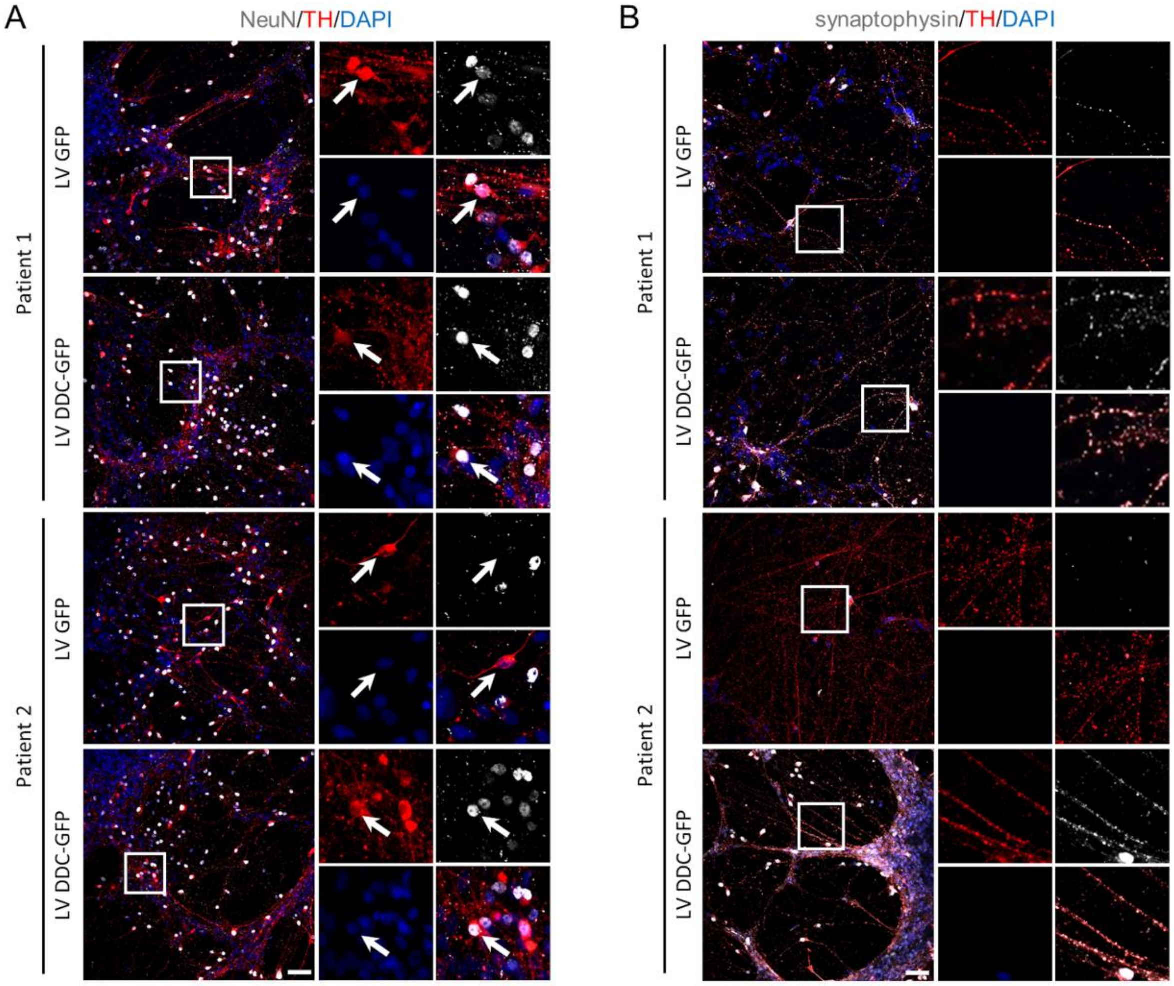

Supplementary Fig. 10: Gene therapy increases NeuN and synaptophysin expression in patient-derived mDA neurons.

(**A**) Immunofluorescence analysis for NeuN and TH in patient-derived neurons transduced with LV GFP and LV DDC-GFP, complete panel from Fig. 6A. Arrows indicate double positive cells. Scale bar 100µm. Inserts show higher magnification of NeuN-positive dopaminergic neurons. (**B**) Representative immunofluorescence images for synaptophysin and TH in patient-derived neurons transduced with LV GFP or LV DDC-GFP, complete panel from Fig. 6D. Scale bar 100µm. Inserts show higher magnification of synaptophysin-positive dopaminergic neurons.

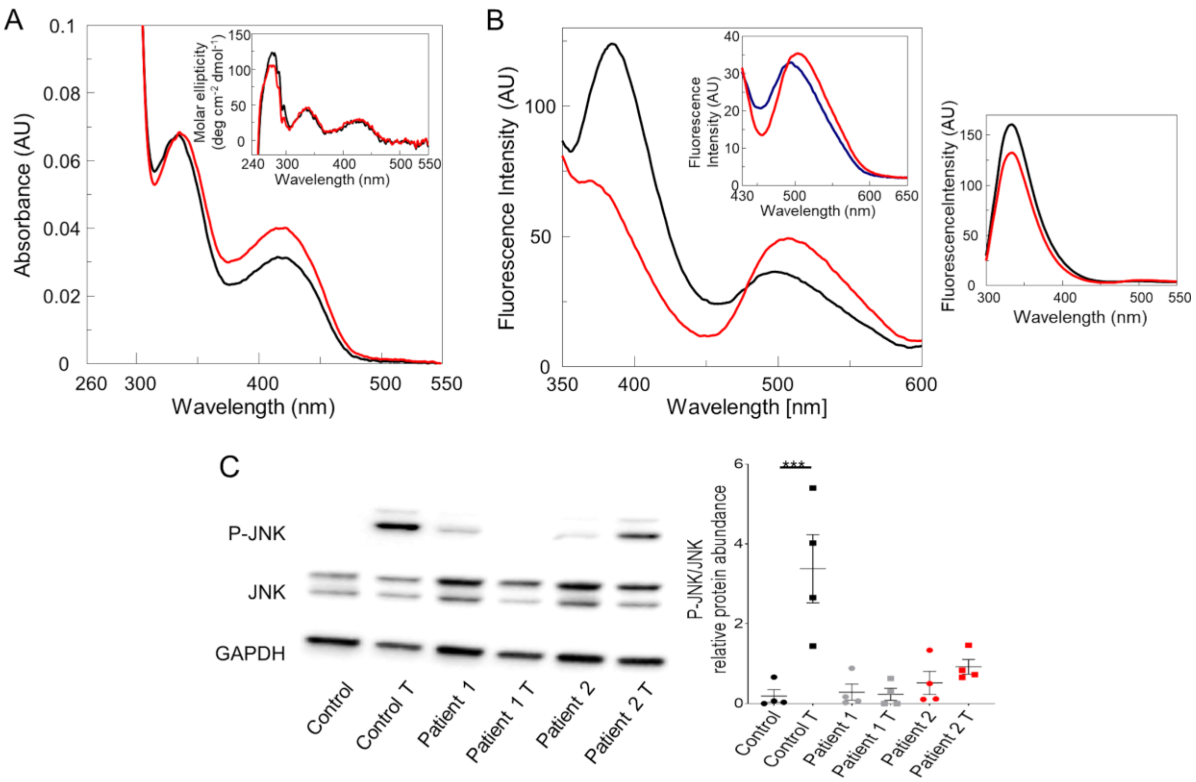

Supplementary Fig. 11: AADCC100S shows no evidence of structural impairment.

(**A**) Absorbance spectra of wild-type AADC and AADCC100S variant. *Inset*) Circular dichroism spectra of wild-type AADC and AADCC100S variant. Spectra were recorded at 7µM enzyme concentration in 100 mM potassium phosphate buffer, pH 7.4. Black: wild-type AADC; Red: AADCC100S. (**B**) Cofactor fluorescence spectra of wild-type AADC and AADCC100S variant at a λ_{ex} 334nm, and *Inset*) Cofactor fluorescence spectra of wild-type AADC and AADCC100S variant at a λ_{ex} 418 nm. Spectra were recorded at 1µM enzyme concentration in 100 mM potassium phosphate buffer, pH 7.4. *Small graph to the right)* Intrinsic fluorescence spectra of wild-type AADC and AADCC100S variant. Spectra were recorded at 0.1µM enzyme concentration in 100 mM potassium phosphate buffer, pH 7.4, at a λ_{ex} 280nm. Black: wild-type AADC; Red: AADCC100S. (**C**) Representative immunoblot for P-JNK, JNK and loading control (GAPDH) and quantification of relative P-JNK/JNK abundance from total cell lysates after L-DOPA treatment (n=4 for all). Data are represented as mean ±SEM. $*P < 0.05$; $**P < 0.01$; $***P < 0.001$, one-way ANOVA followed by Tukey’s multiple comparisons test.

Supplementary Tables:

Supplementary Table 1: Stability, kinetic parameters and PLP affinity of AADC variants.

**Enzyme**	T_m (°C)	$T_{midpoint}$ (°C)	k_{cat} (s^{-1})	K_M (mM)	k_{cat}/K_M ($s^{-1}mM^{-1}$)	$K_{D(PLP)}$ (nM)
AADC [15]	67.1 ± 0.1	56.9 ± 0.6	7.6 ± 0.1	0.11 ± 0.01	69 ± 6	43 ± 12
AADCR347G [15]	67.2 ± 0.1	56.7 ± 0.4	0.016 ± 0.001	0.64 ± 0.13	0.025 ± 0.005	178 ± 14
AADCC100S	67.36 ± 0.03	56.5 ± 0.7	6.9 ± 0.6	0.42 ± 0.10	16 ± 4	112 ± 14

Supplementary Table 2: List of antibodies.

**Name**	**Use, concentration**	**Source, catalogue number**
β-ACT	IB 1:5000	Sigma-Aldrich, A1978
AADC	ICC 1:500	Millipore, AB1569
	IB 1:3000	Cell Signaling Tech, D6N8N
FOXA2	ICC 1:500	BD Pharmigen, 561580
GAPDH-HRP	IB 1:3000	Cell Signaling Tech, 3683
GFP	ICC 1:500	Invitrogen, A6455
JNK	IB 1:1000	Cell Signaling Tech, 9252S
LMX1A	ICC 1:5000	Millipore, AB10533
MAP2	ICC 1:400	Sigma-Aldrich, M9942
NANOG	ICC 1:500	Millipore, MABD24
NeuN	ICC 1:100	Millipore, MAB377
OCT3/4	ICC 1:50	Santa Cruz Biotech, sc-5279
P-JNK	IB 1:1000	Cell Signaling Tech, 9255S
SMA	ICC 1:100	Abcam,
synaptophysin	ICC/IB 1:500/1:2000	Sigma-Aldrich, SAB4502906
SOX17	ICC 1:400	R&D Systems, AF1924
TH	ICC 1:400	Aves Labs, TYH
	IB 1:3000	Millipore, AB152
TPH2	ICC 1:100	Novus Biological, NB100-74555
TRA-1-60	ICC 1:200	Santa Cruz Biotech, sc21705
TRA-1-81	ICC 1:200	Millipore, MAB4381
TUJ1	ICC, 1:400	Biolegend, MMS-435P

ICC: immunocytochemistry, IB: immunoblotting.

Supplementary Table 3: List of primers for RT-PCR and qRT-PCR.

Name	Primer forward sequence	Primer reverse sequence	Use
ALB	TTTGCAGATGTCAGTGAAAGAGA	TGGGGAGGCTATAGAAAATAAGG	qRT-PCR
c-MYC	GCGTCCTGGGAAGGGAGATCCGGAGC	TTGAGGGGCATCGTCGCGGGAGGCTG	RT-PCR
DDC	CCCTACTTCTTCGCCTACTTCC	CACAGTCTCCAGCTCTGTGC	qRT-PCR
EN1	CGTGGCTTACTCCCCATTTA	TCTCGCTGTCTCTCCCTCTC	qRT-PCR
EN2	CCTCCTGCTCCTCCTTTCTT	GACGCAGACGATGTATGCAC	qRT-PCR
ESG1	ATATCCCGCCGTGGGTGAAAGTTC	ACTCAGCCATGGACTGGAGCATCC	RT-PCR
FOXA2	CCGTTCTCCATCAACAACCT	GGGGTAGTGCATCACCTGTT	qRT-PCR
GAPDH	ATCCCATCACCATCTTCCAG	CCATCACGCCACAGTTTCC	RT-PCR
	TTGAGGTCAATGAAGGGGTC	GAAGGTGAAGGTCGGAGTCA	qRT-PCR
LMX1A	CGCATCGTTTCTTCTCCTCT	CAGACAGACTTGGGGCTCAC	qRT-PCR
LMX1B	CTTAACCAGCCTCAGCGACT	TCAGGAGGCGAAGTAGGAAC	qRT-PCR
NANOG	CAGCCCCGATTCTTCCAGTCCC	CGGAAGATTCCCAGTCGGGTTCACC	RT-PCR
	TTGGGACTGGTGGAAGAATC	GATTTGTGGGCCTGAAGAAA	qRT-PCR
NURR1	TCGACATTTCTGCCTTCTCCTG	GGTTCCTTGAGCCCGTGTCT	qRT-PCR
OCT3/4	CGAAACCCACACTGCAGCAG	CCTGGCACAAACTCCAGGTTT	RT-PCR
	TCTCCAGGTTGCCTCTCACT	GTGGAGGAAGCTGACAACAA	qRT-PCR
PITX3	GAGCTAGAGGCGACCTTCC	CCGGTTCTTGAACCACACCC	qRT-PCR
SeV c-MYC	TAACTGACTAGCAGGCTTGTCG	TCCACATACAGTCCTGGATGATGATG	RT-PCR
SeV KLF4	TTCCTGCATGCCAGAGGAGCCC	AATGTATCGAAGGTGCTCAA	RT-PCR
SeV SOX2	ATGCACCGCTACGACGTGAGCGC	AATGTATCGAAGGTGCTCAA	RT-PCR
SeV OCT4	CCCGAAAGAGAAAGCGAACCAG	AATGTATCGAAGGTGCTCAA	RT-PCR
SNCA	GGAGTGGCCATTCGACGAC	CCTGCTGCTTCTGCCACAC	qRT-PCR
SOX2	GGGAAATGGGAGGGGTGCAAAAGAGG	TTGCGTGAGTGTGGATGGGATTGGTG	RT-PCR
TH	CGGGCTTCTCGGACCAGGTGTA	CTCCTCGGCGGTGTACTCCACA	qRT-PCR
WPRE	GTCCTTTCCATGGCTGCTC	CCGAAGGGACGTAGCAGA	qRT-PCR
