# Supplementary material for: Aromatic l-amino acid decarboxylase deficiency: a patient-derived neuronal model for precision therapies
Source: Brain. 2021 Mar 18;144(8):2443–56. doi: 10.1093/brain/awab123 (PMC8418346; doi:10.1093/brain/awab123)
Supplement: awab123_Supplementary_Data [file awab123_supplementary_data.zip › awab123-suppl_data/OP-BRAI210122_PECorr_CmtAttachmentsFolder_Supplementary Data_Full-length blots.pdf]

# Aromatic L-Amino Acid Decarboxylase Deficiency:

# A Patient-derived Neuronal Model for Precision Therapies

Giada Rossignoli[1,2], Karolin Krämer[1], Eleonora Lugarà[3], Haya Alrashidi[4], Simon Pope[5], Carmen De La Fuente Barrigon[4], Katy Barwick[1], Giovanni Bisello[2], Joanne Ng[1,6], John Counsell[1], Gabriele Lignani[3], Simon J. R. Heales[5,7], Mariarita Bertoldi[2,*], Serena Barral[1], Manju A. Kurian[1,8,*]

## Affiliations

1. Developmental Neurosciences, GOS Institute of Child Health, University College London, London WC1N 1EH, UK
2. Biological Chemistry, NBM Department, University of Verona, 37134 Verona, Italy
3. Clinical and Experimental Epilepsy, Queen Square Institute of Neurology, University College London, London WC1N 3BG, UK
4. Genetics and Genomic Medicine, GOS Institute of Child Health, University College London, London WC1N 1EH, UK
5. Neurometabolic Unit, National Hospital for Neurology and Neurosurgery, Queen Square, London WC1N 3BG, UK
6. Gene Transfer Technology Group, EGA-Institute for Women's Health, University College London, London WC1E 6HU, UK
7. Centre for Inborn Errors of Metabolism, GOS Institute of Child Health, University College London, London WC1N 1EH, UK
8. Department of Neurology, Great Ormond Street Hospital, London WC1N 3JH, UK

## *Correspondence to:

Prof Manju Kurian; Zayed Centre for Research, UCL Great Ormond Street Institute of Child Health, 20 Guilford St, London WC1N 1DZ, UK. Email: manju.kurian@ucl.ac.uk

Prof Mariarita Bertoldi; Department of Neuroscience, Biomedicine and Movement Sciences, Biological Chemistry Section, Room 1.24, Strada le Grazie 8, 37134 Verona, Italy. E-mail: mita.bertoldi@univr.it

# Supplementary full-length blots:

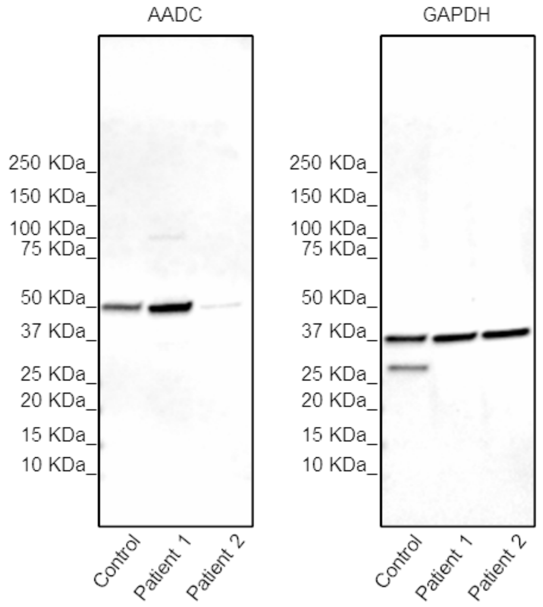


**Supplementary Blot 1: Full-length western blot from Figure 1C.**

Representative immunoblot for AADC and loading control (GAPDH) from patient-derived neuronal cultures.

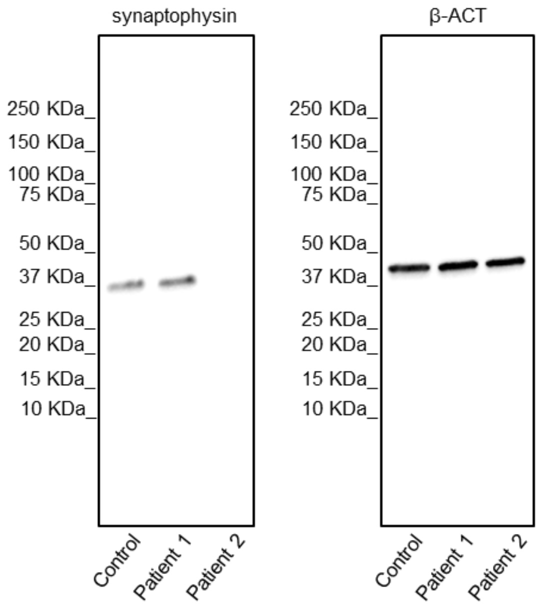


**Supplementary Blot 2: Full-length western blot from Figure 2C.**

Representative immunoblot for synaptophysin and loading control (β-ACT) from patient-derived neuronal cultures.

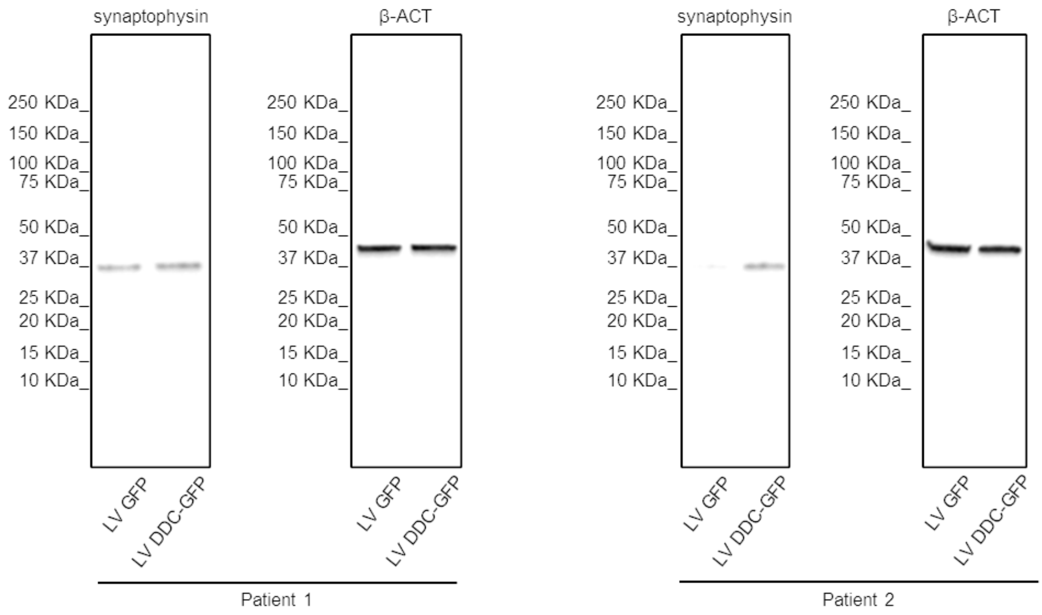


**Supplementary Blot 3: Full-length western blot from Figure 6C.**

Representative immunoblot for synaptophysin and loading control (β-ACT) from LV GFP and LV DDC-GFP transduced patient-derived neuronal cultures.

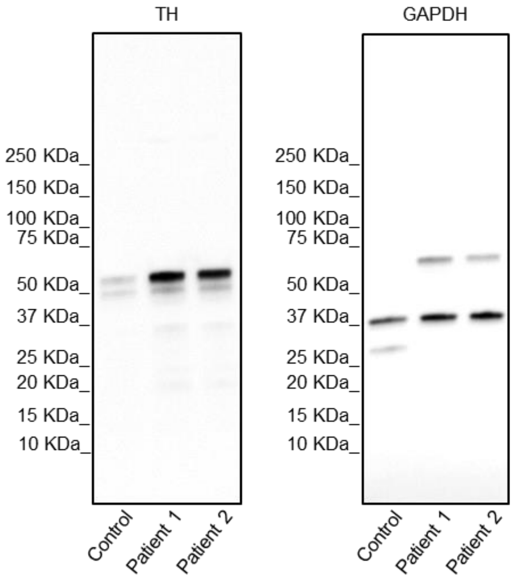


**Supplementary Blot 4: Full-length western blots from Supplementary Figure 5D.**

Representative immunoblot for TH and loading control (GAPDH) from patient-derived neuronal cultures.

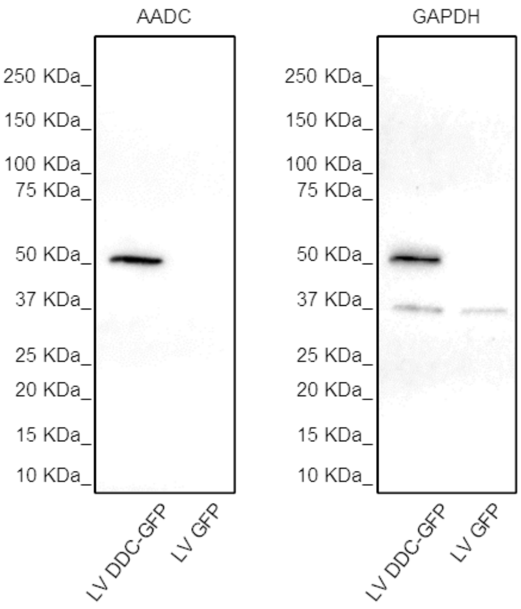


**Supplementary Blot 5: Full-length western blot from Supplementary Figure 8B.**

Representative immunoblot for AADC and loading control (GAPDH) from total lysate of HEK 293T cells 7 days after transfection with LV GFP and LV DDC-GFP.

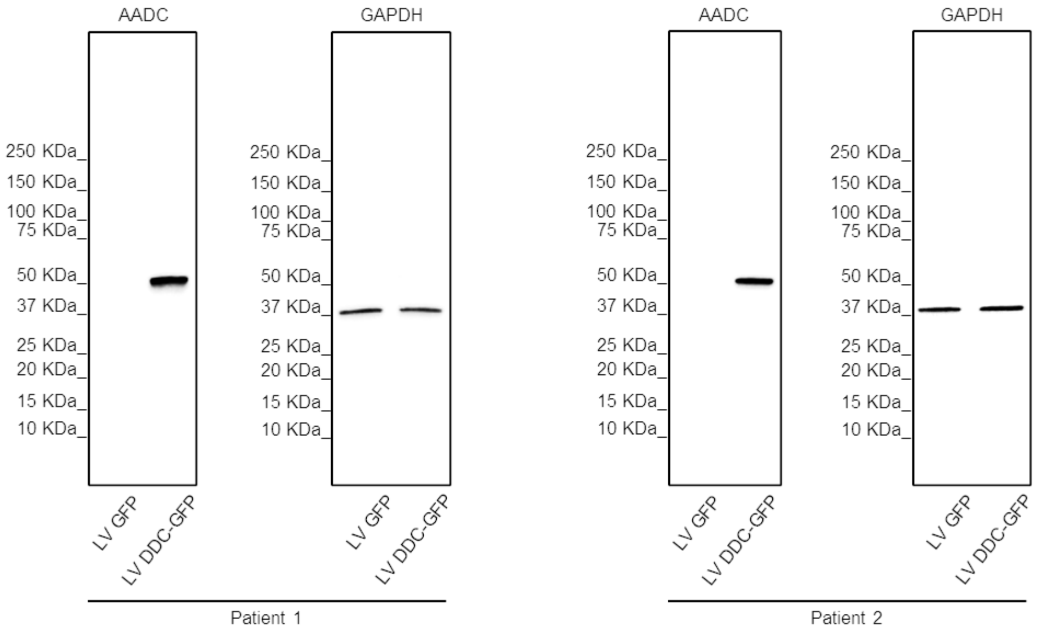


**Supplementary Blot 6: Full-length western blot from Figure 9B.**

Representative immunoblot for AADC and loading control (GAPDH) from LV GFP and LV DDC-GFP transduced patient-derived neuronal cultures.

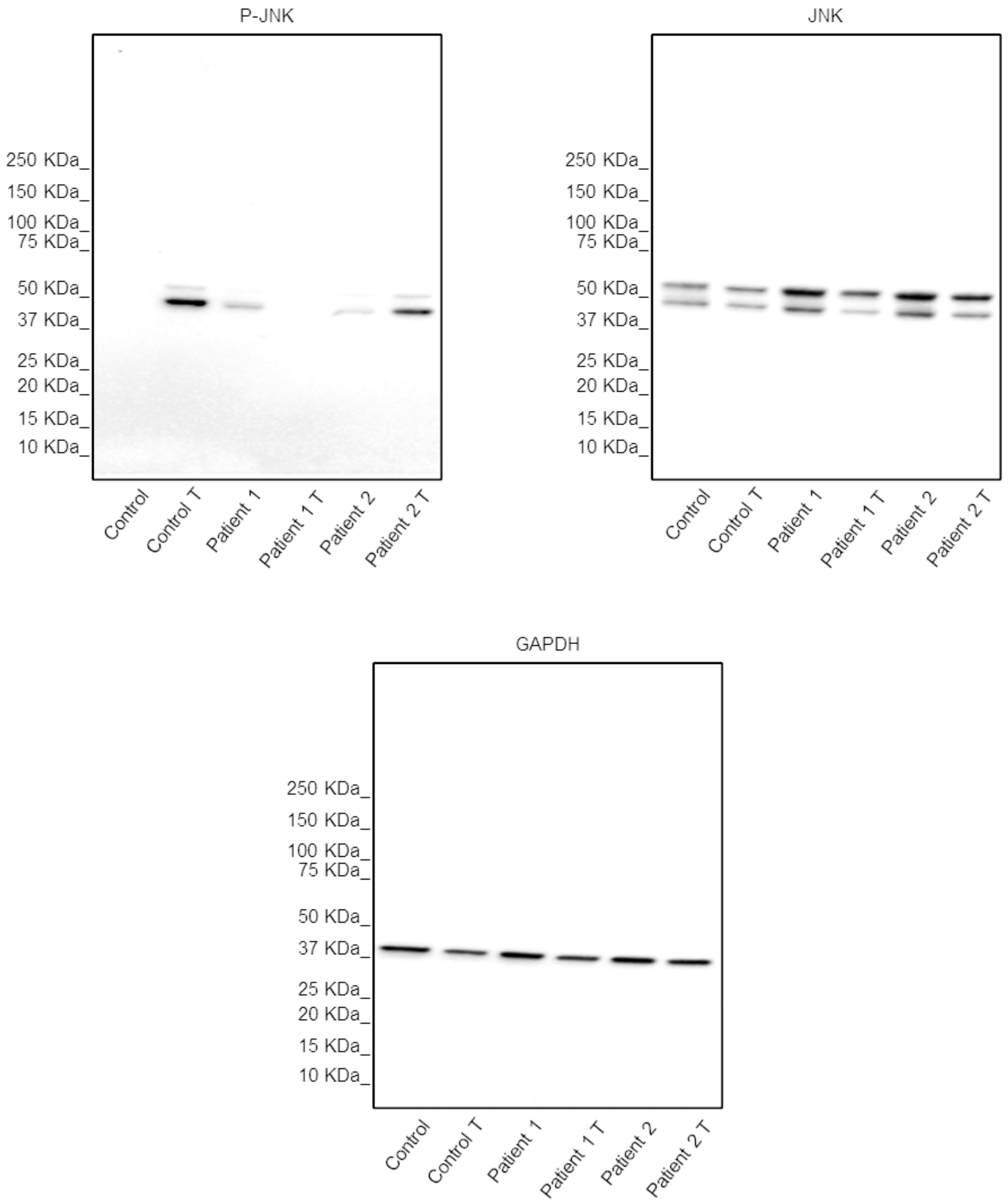


**Supplementary Blot 7: Full-length western blot from Supplementary Figure 11C.**

Representative immunoblot for P-JNK, JNK and loading control (GAPDH) after L-DOPA treatment of patient-derived neuronal cultures.
