# Supplementary material for: Aromatic l-amino acid decarboxylase deficiency: a patient-derived neuronal model for precision therapies
Source: Brain. 2021 Mar 18;144(8):2443–56. doi: 10.1093/brain/awab123 (PMC8418346; doi:10.1093/brain/awab123)
Supplement: awab123_Supplementary_Data [file awab123_supplementary_data.zip › awab123-suppl_data/OP-BRAI210122_PECorr_CmtAttachmentsFolder_Supplementary Data_Materials and Methods.pdf]

Aromatic L-Amino Acid Decarboxylase Deficiency:

A Patient-derived Neuronal Model for Precision Therapies

Giada Rossignoli[1,2], Karolin Krämer[1], Eleonora Lugarà[3], Haya Alrashidi[4], Simon Pope[5], Carmen De La Fuente Barrigon[4], Katy Barwick[1], Giovanni Bisello[2], Joanne Ng[1,6], John Counsell[1], Gabriele Lignani[3], Simon J. R. Heales[5,7], Mariarita Bertoldi[2,*], Serena Barral[1], Manju A. Kurian[1,8,*]

Affiliations

1. Developmental Neurosciences, GOS Institute of Child Health, University College London, London WC1N 1EH, UK
2. Biological Chemistry, NBM Department, University of Verona, 37134 Verona, Italy
3. Clinical and Experimental Epilepsy, Queen Square Institute of Neurology, University College London, London WC1N 3BG, UK
4. Genetics and Genomic Medicine, GOS Institute of Child Health, University College London, London WC1N 1EH, UK
5. Neurometabolic Unit, National Hospital for Neurology and Neurosurgery, Queen Square, London WC1N 3BG, UK
6. Gene Transfer Technology Group, EGA-Institute for Women's Health, University College London, London WC1E 6HU, UK
7. Centre for Inborn Errors of Metabolism, GOS Institute of Child Health, University College London, London WC1N 1EH, UK
8. Department of Neurology, Great Ormond Street Hospital, London WC1N 3JH, UK

*Correspondence to:

Prof Manju Kurian; Zayed Centre for Research, UCL Great Ormond Street Institute of Child Health, 20 Guilford St, London WC1N 1DZ, UK. Email: manju.kurian@ucl.ac.uk

Prof Mariarita Bertoldi; Department of Neuroscience, Biomedicine and Movement Sciences, Biological Chemistry Section, Room 1.24, Strada le Grazie 8, 37134 Verona, Italy. E-mail: mita.bertoldi@univr.it

Supplementary Materials and Methods:

DDC direct Sanger Sequencing in generated iPSCs

DNA from all iPSC lines was extracted using a commercially available kit (DNeasy Blood & Tissue kit, Qiagen), according to manufacturer's instructions. Direct Sanger Sequencing of genomic DNA extracted from control and patient-derived iPSCs was performed to confirm genotype. Genomic DNA sequences were obtained from Alamut® Visual 2.11 software (Genome Reference Consortium Human Build 37 (GRCh37), chromosome 7: 50,458,436-50,565,457; NM_000790.3). Primers were designed with Primer3Plus software (http://www.bioinformatics.nl/cgi-bin/primer3plus/primer3plus.cgi). Primer sequences and PCR conditions are available on request. Amplified DNA was then purified with MicroCLEAN Kit (Clent Life Science) and sequenced in both forward and reverse directions using BigDye® Terminator v1.1 Cycle Sequencing Kit (Thermo Fisher Scientific) on an ABI PRISM 3730 DNA Analyzer (Applied Biosystems). The results were then analysed using Sequencher (https://www.genecodes.com) and Chromas software (http://technelysium.com.au/wp/chromas).

Karyotyping with single-nucleotide polymorphism (SNP) Array

Genome integrity was assessed through SNP array analysis, undertaken by UCL Genomics on genomic DNA extracted from iPSCs, using Infinium HumanCytoSNP-12 v2.1 BeadChip array and analysed with Bluefuse Multi software (Illumina).

Analysis of pluripotency by Epi-Pluri-Score

All derived iPSC lines were analyzed using Illumina HumanMethylation27 BeadChip platform calculating Epi-Pluri-Score (Cygenia), based on the combination of DNA methylation levels at the two CpG sites of *ANKRD46* and *C14orf115*.[1]

Analysis of pluripotency by *in vitro* spontaneous differentiation

iPSCs were harvested with TrypLE™ and plated onto non-adherent bacterial dishes at a concentration of 2×10^6 cells per cm^2 to generate EBs in KO-DMEM, 20% serum replacement, 2 Mm L-glutamine, 50 µM 2-mercaptoethanol, 1% MEM non-essential amino acids, 1% P/S, 1 µM ROCK-inhibitor (Thiazovivin, Cambridge Bioscience). For differentiation into ectoderm and endoderm, EBs were plated at Day 4 on matrigel-coated dishes and maintained until Day 16 in EBs, without ROCK-inhibitor. Mesoderm differentiation was achieved after plating EBs

onto 0,1% galantine (Sigma-Aldrich) coated dishes in DMEM, 20% fetal bovine serum, and 2 mM L-glutamine for 16 days.

Immunocytochemistry

Cells were fixed in 4% paraformaldehyde. Immunofluorescence for OCT3/4, NANOG, SOX17, SMA, TUJ1, FOXA2 and LMX1A was performed in 0.1% triton X-100 and 10% fetal bovine serum in 1x phosphate buffered saline; for TRA-1-60 and TRA-1-81, triton X-100 was omitted. Immunostaining of samples at Day 65 of differentiation was performed in the same buffer solution with 0.3% triton. After blocking for 30 min at room temperature, all primary antibodies (Supplementary Table 2) were incubated overnight at 4°C. Cells were then washed three times with 1x phosphate buffered saline and incubated with the respective species-specific secondary antibodies Alexa 488, Alexa 594 or Alexa 633 (Invitrogen), for 45 min at room temperature. Nuclei were stained with DAPI for 5 min at room temperature, and coverslips were mounted with ProLong Gold Antifade Mountant (Invitrogen). Imaging was performed with Olympus IX71 inverted TC scope for assessment of pluripotency in iPSCs, spontaneous *in vitro* differentiation of iPSCs, and Day 11 mDA precursors. A multiphoton confocal microscope (Zeiss LSM880) was used for all other immunocytochemistry studies. For total quantification, 3 random fields were imaged from each independent experiment, and 1800 total randomly selected nuclei were counted using ImageJ software (National Institutes of Health). Primary neurite branching was quantified as an average from 150 dispersed nuclei in random fields from at least 3 different biological replicates.

Reverse transcription PCR (RT-PCR) and Quantitative Real Time PCR (qRT-PCR) Analysis

RNA was purified from cells using the RNeasy mini kit (Qiagen) following manufacturer instructions. Contaminating genomic DNA was removed from total RNA (1 µg) using DNAseI purification kit (Invitrogen), followed by cDNA generation by reverse transcription using Superscript III (Invitrogen). Sendai virus clearance PCR was performed using manufacturer recommended primers (Invitrogen) (Supplementary Table 3) and protocol. PCR for assessing pluripotency was done using specific oligos reported in Supplementary Table 3.

qRT-PCR analysis was performed using StepOnePlus™ Real-Time PCR System (Applied Biosystems). Reaction mix was prepared with 1x MESA Blue qPCR MasterMix Plus for SYBR® Assay (Eurogentec), 1:25 cDNA and 500 nM of each primer (Supplementary Table 3). All reactions were performed in technical triplicates with the following protocol:

denaturation of 95°C for 5 min, followed by 40 cycles of 15 seconds denaturation at 95°C and 1 min annealing/extension at 60°C. Relative quantification of gene expression was determined using the $2^{-\Delta\Delta Ct}$ method with *GAPDH* as housekeeping reference gene, and normalized to the corresponding iPSCs.

Total protein quantification

Proteins were extracted from cells in ice-cold radioimmunoprecipitation assay lysis and extraction buffer (Sigma-Aldrich) supplemented with protease-phosphatase inhibitor cocktail at the manufacturer recommended concentration. Cells were then incubated on ice for 30 min and centrifugated at 13.000×*g* for 15 min. Total proteins in collected supernatant were quantified with Pierce™ BCA Protein Assay Kit (Thermo Fisher Scientific) using provided standards.

Immunoblotting

10 or 20 µg of total proteins were denatured in 1x Laemmli buffer (Bio-Rad Laboratories LTD) and 100 mM dithiothreitol. Proteins were separated on 4-20% Mini-PROTEAN® TGX™ Stain free Protein Gel (Bio-Rad) and transferred to Trans-Blot® Turbo™ Mini PVDF Transfer membrane using Trans-Blot® Turbo™ Transfer System (Bio-Rad). After blocking in 5% milk in tris-buffered saline with 0.1% Tween for 1h at room temperature, membranes were incubated with primary antibodies (Supplementary Table 2) diluted in 1% milk in tris-buffered saline with 0.1% Tween overnight at 4°C. Membranes were then incubated with the proper secondary horseradish peroxidase-conjugated antibody (Cell Signalling) at a dilution of 1:3000 in 1% milk in tris-buffered saline with 0.1% Tween for 1h at room temperature. Membranes were visualized with Chemidoc MP (Bio-Rad Laboratories). The intensity of bands was quantified using ImageJ software (National Institutes of Health) and normalized to GAPDH or β-ACT after membranes clearance with Restore Western Blot Stripping Buffer (Thermo Scientific) and re-probing.

Lentivirus generation and gene transfer

The lentiviral expression plasmid (pCCL-hSYN1-DDC-IRES-EGFP) was developed by inserting the human *DDC* gene coding sequence (NM_000790.3) into a previously developed plasmid, kindly provided by Dr Joanne Ng (UCL Institute of Women's Health), who also produced the control plasmid CCL-hSYN1-GFP. Plasmids sequences were verified by Sanger sequencing; primer sequences and PCR conditions are available on request. Lentiviral expression plasmid validation was performed by transducing HEK 293T cells and performing

immunoblotting for AADC 72 h later. Lentiviral vectors were produced using a 2nd generation packaging system[2] and vector titers were quantified by qPCR as previously described[3] using primers listed in Supplementary Table 3. mDA neurons were differentiated for 24 days before transduction with lentiviral vectors at 0.5 multiplicity of infection for 24 h, and subsequently differentiated, as previously described, to Day 65.

Protein bioinformatics and biochemical analyses

The structure corresponding to *sus scrofa* holoenzyme solved in complex with PLP and carbidopa (PDB code: 1JS3) was visualized and analysed using PyMol™ software (1.7.4.5. Edu version). Recombinant human AADC variants were obtained by site-specific mutagenesis using the template DNA previously published,[4] and Quick-Change II kit (Agilent technologies), and the specific nucleotide change was confirmed by sequencing. Primer sequences and PCR conditions are available on request. Expression, purification, spectroscopic characterization and parameters calculation analyses were performed as previously reported.[4–6]

References

1. Lenz M, Goetzke R, Schenk A, et al. Epigenetic biomarker to support classification into pluripotent and non-pluripotent cells. *Sci Rep*. 2015; 5:8973. doi:10.1038/srep08973.

2. Counsell JR, Asgarian Z, Meng J, et al. Lentiviral vectors can be used for full-length dystrophin gene therapy. *Sci Rep*. 2017; 7:44775. doi:10.1038/srep44775.

3. Barczak W, Suchorska W, Rubiś B, Kulcenty K. Universal Real-Time PCR-Based Assay for Lentiviral Titration. *Mol Biotechnol*. 2014; 57(2):195-200. doi:10.1007/s12033-014-9815-4.

4. Montioli R, Dindo M, Giorgetti A, Piccoli S, Cellini B, Voltattorni CB orr. A comprehensive picture of the mutations associated with aromatic amino acid decarboxylase deficiency: from molecular mechanisms to therapy implications. *Hum Mol Genet*. 2014; 23(20):5429-5440. doi:10.1093/hmg/ddu266.

5. Montioli R, Paiardini A, Kurian MA, et al. The novel R347g pathogenic mutation of aromatic amino acid decarboxylase provides additional molecular insights into enzyme catalysis and deficiency. *Biochim Biophys Acta - Proteins Proteomics*. 2016; 1864(6):676-682. doi:10.1016/j.bbapap.2016.03.011.

6. Montioli R, Bisello G, Dindo M, Rossignoli G, Voltattorni CB, Bertoldi M. New variants of AADC deficiency expand the knowledge of enzymatic phenotypes. *Arch Biochem Biophys*. 2020; 682:108263. doi:10.1016/j.abb.2020.108263
